# *Streptococcus suis* serotype 5: Emerging zoonotic threat with distinct genomic heterogeneity

**DOI:** 10.1080/21505594.2025.2523882

**Published:** 2025-06-26

**Authors:** Xiyan Zhang, Jinlu Zhu, Anusak Kerdsin, Jianping Wang, Mingliu Wang, Hui Yang, Weiming Kang, Xiaojing Lu, Yan Wang, Hui Sun, Marcelo Gottschalk, Han Zheng, Jianguo Xu, Zongfu Wu

**Affiliations:** a National Institute for Communicable Disease Control and Prevention, Chinese Center for Disease Control and Prevention, Changping, Beijing, China; b WOAH Reference Lab for Swine Streptococcosis, College of Veterinary Medicine, Nanjing Agricultural University, Nanjing, China; c Faculty of Public Health, Kasetsart University, Chalermphrakiat Sakon Nakhon Province Campus, Sakon Nakhon, Thailand; d Guangxi Zhuang Autonomous Region Center for Disease Prevention and Control, Acute Infectious Disease Prevention and Control Institute, Guangxi Zhuang Autonomous Region, Nanning, China; e Nanning Center for Disease Control and Prevention, Guangxi Zhuang Autonomous Region, Nanning, China; f Central Laboratory, Tongji Hospital, School of Medicine, Tongji University, Shanghai, China; g Swine and Poultry Infectious Diseases Research Center, Faculty of Veterinary Medicine, University of Montreal, Quebec, Canada; h Department of Biochemistry and Molecular Biology, Shanxi Key Laboratory of Birth Defect and Cell Regeneration, MOE Key Laboratory of Coal Environmental Pathogenicity and Prevention, Shanxi Medical University, Taiyuan, China; i National Key Laboratory of Intelligent Tracking and Forecasting for Infectious Diseases, Changping, Beijing, China

**Keywords:** *Streptococcus suis*, serotype 5, population structure, zoonotic potential, pathogenicity

## Abstract

*Streptococcus suis* is a significant pig pathogen and an emerging zoonotic agent. Serotype 5 is becoming an increasing concern among pigs and humans with *S. suis* infection worldwide. This study investigated the population structure, phylogenetic relationship, genomic characteristics, and virulence of serotype 5 population, analyzing 89 isolates, including eight from human cases. The results revealed significant genomic heterogeneity and diverse virulence levels within serotype 5 population. Phylogenetic analysis identified two distinct lineages with notable differences in evolution and genomic traits. Thirty-two representative serotype 5 strains were clustered into four groups: ultra-highly virulent (UV) (*n* = 1), highly virulent plus (HV^+^) (*n* = 4), HV (*n* = 11), and virulent (V) (*n* = 16). Virulence levels progressively decreased from the UV group to HV^+^, HV, and V groups. The UV, HV^+^, and HV strains induced significantly lethal infection in mice during the early phase of infection. The lethal infection induced by UV and HV^+^ strains was time-dependent but dose-independent. Ultra-high bacterial loads, excessive pro-inflammatory cytokines, and severe organ damage were responsible for the sudden death of mice infected with UV strain at the early phase of infection. The capacity to establish infection, induce excessive pro-inflammatory cytokine production, and elevate biomarkers associated with organ damage varied significantly among HV^+^ strains. The V strains demonstrated the capacity to induce delayed lethal infection. These findings emphasized the serious public health risk posed by serotype 5 strains. The valuable information for developing effective prevention and control strategies for *S. suis* serotype 5 infections was provided.

## Introduction

*Streptococcus suis* (*S. suis*) is a significant pathogen in pigs and an emerging zoonotic agent capable of causing meningitis, septicemia, arthritis, pneumonia, endocarditis, shock, and even death in both pigs and humans worldwide [1–3]. Human *S. suis* infections have become endemic in Asian countries such as Thailand, Vietnam, Indonesia, and China [3–5]. Furthermore, *S. suis* has caused multiple outbreaks in China and Thailand [6,7]. The consumption of *S. suis* contaminated raw or undercooked pork, blood, and offal products plays a crucial role in *S. suis* infection in Thailand, Vietnam, and Indonesia [1,4,8]. In America, Europe, and China, human *S. suis* cases are usually related to occupational exposure via skin injuries [1,9]. Serotyping based on capsular polysaccharide antigens or *cps*-specific *wzy* genes is essential for diagnosing and epidemiological studies of *S. suis*. A total of 29 serotypes (1–19, 21, 23–25, 27–31, and 1/2) [10,11] and 34 novel *cps* loci [7,12] based on the *cps*-specific *wzy* genes in *S. suis* have been identified. Human *S. suis* cases with serotypes 1, 2, 4, 5, 7, 9, 14, 16, 21, 24, and 31 have been reported [2,13–22]. Although serotype 2 is the most prevalent among pigs and humans with *S. suis* infection worldwide [2], serotype 5 is increasingly concerning. *S. suis* serotype 5 strains were routinely isolated from diseased pigs worldwide [9,23–27]. The first human *S suis* infection with serotype 5 caused by eating raw pork was reported in Thailand in 2007 [28]. Prior to this report, 12 human cases of *S. suis* infection caused by serotype 5 strains have been reported, with cases distributed across the USA (*n* = 1) [29], Argentina (*n* = 1) [30], Thailand (*n* = 4) [13], Sweden (*n* = 1) [31], Japan (*n* = 1) [32], and China (*n* = 4). Of the cases in China, two were reported in references [33,34], and the other two were reported by the Chinese Pathogen Identification Net in 2022 and 2023, respectively. In the present study, we isolated an *S. suis* serotype 5 strain GX169 from the peripheral blood culture of a Streptococcal toxic shock-like syndrome (STSLS) patient in Guangxi Zhuang Autonomous Region (GX) in 2021. This indicates that the threat of *S. suis* serotype 5 strains to public health is increasing worldwide. To date, the understanding of the epidemiology and pathogenicity of *S. suis* serotype 5 remains limited. Investigating its potential pathogenicity for both humans and pigs is an urgent imperative.

This study investigated the population structure and genomic characteristics of *S. suis* serotype 5 strains isolated from a wide range of hosts, including human patients, diseased pigs, and healthy pigs. The virulence levels of selected representative strains were evaluated. The underlying mechanisms contributing to the increased pathogenicity of serotype 5 strains were further examined.

## Methods and materials

### Human case description

In 2021, a 45-year-old male chef specializing in pork processing was admitted and was diagnosed with STSLS. The characteristic laboratory tests results included platelet count (66 × 10^9^/L), high-sensitivity C-reactive protein (134.23 mg/L), thrombin time (TT, 18.9 s), prothrombin time (PT, 17.0 s) of, activated partial thromboplastin time (APTT, 48.9 s), alanine transaminase (ALT, 440 U/L), aspartate transaminase (AST, 426 U/L), creatinine (CR, 442.6 μmol/L), and lactate dehydrogenase (LDH, 713 U/L). The patient’s condition significantly improved after antibiotic therapy (a combination of piperacillin sodium, tazobactam sodium, and levofloxacin) and supportive care. The patient was discharged 10 days after admission.

### Bacterial strains, sequencing, and bioinformatic analysis

In total, 43 genomes (four from human cases) from NCBI and 46 strains from this study were included (Table 1). Four of 46 strains were from human cases. The strain GX169 was provided by RA Hui Yang, whereas the strains ID48908, ID24665, and ID41570 were provided by Professor Anusak Kerdsin. Forty-two of 46 strains were isolated from pigs. The serotype 5 reference strain 11,538 was provided by Professor Marcelo Gottschalk. Remaining 41 strains were isolated by Professor Han Zheng and Zongfu Wu, respectively.

**Table 1.** The information of *S. suis* serotype 5 strains and genomes used in the study.

Lineage		Name of strains	MCG	ST	*cps* type	Country	Collection date	Host	Isolation	Type	GenBank assembly accession
Lineage 1	Lineage 1–1	EJ2T3-2B	2	2246	Ia-1	Canada	2016	Pig	/	NCBI Genome	SAMN14932579
		EJ2T3-1B	2	2246	Ia-1	Canada	2016	Pig	/	NCBI Genome	SAMN14932577
		EJ2T3-1A	2	2246	Ia-1	Canada	2016	Pig	/	NCBI Genome	SAMN14932576
		1607744	2	89	Ia-1	Canada	2014	Pig	/	NCBI Genome	SAMN14932478
		TMW_SS074	2	87	Ib-1	United Kingdom	2014	Pig	/	NCBI Genome	SAMN14933254
		TMW_SS065	2	87	Ib-1	United Kingdom	2014	Pig	/	NCBI Genome	SAMN14933247
	Lineage 1–2	40440	3	54	Ia-1	USA	2016	Pig	/	NCBI Genome	SAMN13975664
		11538	3	53	Ia-1	Netherlands	1980s-1990s	Diseased Pig	/	Strain	SAMN02470634
		GX169	3	2249	Ia-1	China	2021	Patient	Blood	Strain^a^	SAMN33923801
		1547095	3	1197	Ia-2	USA	2014	Patient	Jointfluid	NCBI Genome	SAMN37410674
		1652329	3	483	Ia-1	Argentina	2014	Patient	Cerebrospinalfluid	NCBI Genome	SAMN37410675
		TANI1	3	752	Ia-1	Japan	2016	Patient	Blood	NCBI Genome	SAMD00066479
		93–1320	3	977	Ib-1	Canada	1993	Pig	/	NCBI Genome	SAMN14932536
		93–2042–4514	3	977	Ib-1	Canada	1993	Pig	/	NCBI Genome	SAMN14932537
		00–3638-4B	3	977	Ib-1	Canada	2000	Pig	/	NCBI Genome	SAMN14932408
		30212	3	977	Ib-1	USA	2015	Pig	/	NCBI Genome	SAMN13975597
		CF2D3-4A	3	1175	Ia-1	Canada	2016	Pig	/	NCBI Genome	SAMN14932554
		1148794	3	1175	Ia-1	Canada	2009	Pig	/	NCBI Genome	SAMN14932415
		CF2D3-2E	3	1175	Ia-1	Canada	2016	Pig	/	NCBI Genome	SAMN14932553
		92–2402–1119	3	1175	Ia-1	Canada	1992	Pig	/	NCBI Genome	SAMN14932535
		1637946	3	94	Ia-1	Canada	2014	Pig	/	NCBI Genome	SAMN14932492
		90–546	3	1175	Ia-1	Canada	1990	Pig	/	NCBI Genome	SAMN14932528
		38728	3	94	Ia-1	USA	2016	Pig	/	NCBI Genome	SAMN13975696
		29898	3	119	Ia-2	USA	2015	Pig	/	NCBI Genome	SAMN13975610
	Lineage 1–3	2020WUSS075	N	2241	Ib-1	China	2020	Healthy pig	Tonsil	Strain^a^	SAMN33923834
		2020WUSS080	N	2241	Ib-1	China	2020	Healthy pig	Tonsil	Strain^a^	SAMN33923835
		ID26102	N	236	Ib-1	Thailand	2008	Healthy pig	/	NCBI Genome	SAMN37410680
		ID48908	N	236	Ib-1	Thailand	2014	Patient	Cerebrospinalfluid	Strain	SAMN37410678
		WUSS266	N	2252	Ib-1	China	2017	Healthy pig	Blood	Strain^a^	SAMN33923823
		684_10A	N	2247	Ib-1	United Kingdom	2013	Pig	/	NCBI Genome	SAMN14933345
Lineage 2	Lineage 2–1	874	6	2245	Ib-2	China	2013	Pig	Tonsil	NCBI Genome	SAMN08295915
		805	6	2245	Ib-2	China	2013	Pig	Tonsil	NCBI Genome	SAMN08295899
		803	6	2245	Ib-2	China	2013	Pig	Tonsil	NCBI Genome	SAMN08295898
		YS157	6	413	Ib-2	China	2012	Healthy pig	Nasopharynx swab	Strain^a^	SAMN33923805
		YS106	6	406	Ib-3	China	2012	Healthy pig	Nasopharynx swab	Strain^a^	SAMN33923840
		YS119	6	406	Ib-3	China	2012	Healthy pig	Nasopharynx swab	Strain^a^	SAMN33923804
		YS395	6	2263	Ib-2	China	2014	Healthy pig	Nasopharynx swab	Strain^a^	SAMN33923814
		2021WUSS081	6	2243	Ib-2	China	2021	Healthy pig	Tonsil	Strain^a^	SAMN33923838
		YS294	6	2261	Ib-2	China	2013	Healthy pig	Nasopharynx swab	Strain^a^	SAMN33923812
		WUSS363	6	2259	Ib-1	China	2017	Healthy pig	Tonsil	Strain^a^	SAMN33923830
		YS307	6	2262	Ib-2	China	2013	Healthy pig	Nasopharynx swab	Strain^a^	SAMN33923813
		YS599	6	2267	Ib-3	China	2015	Healthy pig	Nasopharynx swab	Strain^a^	SAMN33923842
		YS608	6	2268	Ib-3	China	2015	Healthy pig	Nasopharynx swab	Strain^a^	SAMN33923819
		YS88	6	2269	Ib-2	China	2011	Healthy pig	Nasopharynx swab	Strain^a^	SAMN33923802
		YS89	6	2269	Ib-2	China	2012	Healthy pig	Nasopharynx swab	Strain^a^	SAMN33923803
		2021WUSS082	6	2244	Ib-4	China	2021	Healthy pig	Tonsil	Strain^a^	SAMN33923839
		JHSJ1	6	2248	Ib-2	China	2016	Diseased pig	/	NCBI Genome	SAMN20953750
		ZKSJ4	6	2248	Ib-2	China	2016	Diseased pig	/	NCBI Genome	SAMN20953776
		WUSS336	6	2256	Ib-3	China	2017	Healthy pig	Tonsil	Strain^a^	SAMN33923827
		YS177	6	2260	Ib-3	China	2012	Healthy pig	Nasopharynx swab	Strain^a^	SAMN33923807
		YS572	6	2266	Ib-3	China	2015	Healthy pig	Nasopharynx swab	Strain^a^	SAMN33923817
		YS580	6	2266	Ib-3	China	2015	Healthy pig	Nasopharynx swab	Strain^a^	SAMN33923818
		YS174	6	474	Ib-3	China	2012	Healthy pig	Nasopharynx swab	Strain^a^	SAMN33923806
		YS242	6	563	Ib-2	China	2013	Healthy pig	Nasopharynx swab	Strain^a^	SAMN33923810
		YS561	6	2265	Ib-3	China	2015	Healthy pig	Nasopharynx swab	Strain^a^	SAMN33923841
		YS188	6	524	Ib-1	China	2013	Healthy pig	Nasopharynx swab	Strain^a^	SAMN33923808
		YS226	6	552	Ib-1	China	2013	Healthy pig	Nasopharynx swab	Strain^a^	SAMN33923809
		YS259	6	575	Ib-1	China	2013	Healthy pig	Nasopharynx swab	Strain^a^	SAMN33923811
		YS468	N	2264	Ib-1	China	2015	Healthy pig	Nasopharynx swab	Strain^a^	SAMN33923815
	Lineage 2–2	2464	7–3	499	Ib-1	China	2013	Pig	Tonsil	NCBI Genome	SAMN08296041
		1371	7–3	499	Ib-1	China	2013	Pig	Tonsil	NCBI Genome	SAMN08295996
		180	7–3	499	Ib-1	China	2013	Pig	Tonsil	NCBI Genome	SAMN08295859
		SH0918	7–3	499	Ib-1	China	2009	Pig	/	NCBI Genome	SAMN20953763
		CPD5	7–3	499	Ib-1	China	2009	Pig	/	NCBI Genome	SAMN12784781
		CPD34	7–3	936	Ib-1	China	2013	Pig	/	NCBI Genome	SAMN12784772
		CPD32	7–3	943	Ib-1	China	2011	Pig	/	NCBI Genome	SAMN12784770
		WUSS276	7–3	2253	Ib-1	China	2017	Healthy pig	Tonsil	Strain^a^	SAMN33923824
		2018WUSS006	7–3	500	Ib-1	China	2018	Healthy pig	Tonsil	Strain^a^	SAMN33923831
		2018WUSS036	7–3	2240	Ib-1	China	2018	Healthy pig	Tonsil	Strain^a^	SAMN33923832
		WUSS354	7–3	2257	Ib-1	China	2017	Healthy pig	Tonsil	Strain^a^	SAMN33923828
		2020WUSS051	7–3	1915	Ib-1	China	2020	Healthy pig	Tonsil	Strain^a^	SAMN33923833
		WUSS289	7–3	2255	Ib-1	China	2017	Healthy pig	Tonsil	Strain^a^	SAMN33923826
		2020WUSS088	7–3	2242	Ib-1	China	2020	Diseased pig	Lung	Strain^a^	SAMN33923837
		WUSS358	7–3	2258	Ib-1	China	2017	Healthy pig	Tonsil	Strain^a^	SAMN33923829
		WUSS281	7–3	2254	Ib-1	China	2017	Healthy pig	Tonsil	Strain^a^	SAMN33923825
		ID24665	7–3	181	Ib-1	Thailand	2007	Patient	Asciticfluid	Strain	SAMN37410676
		ID32563	7–3	235	Ib-1	Thailand	2010	Patient	Blood	NCBI Genome	SAMN37410677
		ID34567	7–3	235	Ib-1	Thailand	2011	Healthy pig	/	NCBI Genome	SAMN37410679
		WUSS225	7–3	2250	Ib-3	China	2017	Healthy pig	Nasopharynx swab	Strain^a^	SAMN33923821
		WUSS233	7–3	2251	Ib-3	China	2017	Healthy pig	Nasopharynx swab	Strain^a^	SAMN33923822
		YS539	7–3	N	Ib-2	China	2015	Healthy pig	Nasopharynx swab	Strain^a^	SAMN33923816
		2017UMN1435.22	7–3	1214	Ib-1	Canada	2017	Healthy pig	Tonsil	NCBI Genome	SAMN11854277
		MA4T3-4A	7–3	1214	Ib-1	Canada	2016	Pig	/	NCBI Genome	SAMN14932590
		MA4T3-4B	7–3	1214	Ib-1	Canada	2016	Pig	/	NCBI Genome	SAMN14932591
		MA4T3-4D	7–3	1214	Ib-1	Canada	2016	Pig	/	NCBI Genome	SAMN14932593
		2020WUSS085	7–3	1671	Ib-1	China	2020	Healthy pig	Tonsil	Strain^a^	SAMN33923836
		ID41570	7–3	221	Ib-1	Thailand	2012	Patient	Blood	Strain	SAMN20090113
		HN105	7–3	498	Ib-1	China	2014	Pig	/	NCBI Genome	SAMN09080446
		WUSS027	7–3	498	Ib-1	China	2017	Healthy pig	/	Strain^a^	SAMN33923820

a: sequenced in the study

The genomes and strains identity as *S. suis* and their classification as serotype 5 was confirmed through analysis of their full-length *16S rRNA* gene sequences [35] and the presence of the serotype 5-specific *wzy* gene (GenBank: FAA00878.1), respectively. The strains were isolated between the 1980s and 2021, with a geographic distribution spanning eight countries: China (*n* = 55), Canada (*n* = 17), Thailand (*n* = 6), the United States (*n* = 5), the United Kingdom (*n* = 3), and one genome each from Argentina, Japan, and the Netherlands.

Among 46 strains from this study, four strains (11538, ID24665, ID41570, and ID48908) were sequenced in previous studies [13,36], and 42 strains were sequenced in the present study according to the methods described in our previous studies [16,36], consisting of the complete genome of strain GX169 and the 41 draft genomes.

The multilocus sequence typing (MLST) and minimum core genome (MCG) typing of each genome were determined by analyzing corresponding whole genome sequences in the PubMLST database (https://pubmlst.org/bigsdb?db=pubmlst_ssuis_seqdef) and the Pathogen Genome and Metagenome Analysis Cloud Platform (https://analysis.mypathogen.org/workflow/config/chinacdc/Ssuis_CGT/1/), respectively.

Each genome general feature format (gff) annotation file was generated using Prokka v1.13 with default parameters [37]. The core-genome of 89 serotype 5 genomes was constructed using the gff annotation files by the Roary pipeline with the following parameters: cd (the percentage of isolates a gene must be present in to be considered core) set to 99 and i (minimum percentage identity for BLASTP) set to 90.

Single-nucleotide polymorphisms (SNPs) in the core genome were detected using MUMmer v3.23. The recombinant SNP sites were removed based on the method described in a previous study [36]. The phylogenetic tree based on the distribution of mutant SNPs in the core genomes was constructed using the maximum likelihood method by FastTree v2.1.10. The genome sequence of SC84 (accession No. FM252031) was used as a reference and *Streptococcus pneumoniae* ATCC 700,669 (accession No. NC_011900) was used as an outgroup to root the tree. The resulting tree was visualized using FigTree v1.4.0.

The distributions of 154 known *S. suis* putative virulence-associated genes in serotype 5 genomes were investigated. The presence of antibiotic resistance (AR) genes was determined by searching the ResFinder database (https://cge.food.dtu.dk/services/ResFinder/). Virulence-associated genes and AR genes exhibiting a global match region below 80% and an amino acid sequence identity below 80% were considered absent [38]. The intact *cps* gene cluster was extracted and compared according to the method described in the previous study [16].

### Analysis of antimicrobial susceptibility and mobile genetic elements (MGEs) harboring the AR genes

The antimicrobial susceptibility profiles of penicillin, cefotaxime, vancomycin, florfenicol, and linezolid were evaluated according to the method and resistance breakpoints according to the methods previously described [39]. The intact MGEs carrying AR genes, including integrative and conjugative elements (ICEs) and prophages integrated into *rpIL* gene (*SSU0845*), *rumA* gene (*SSU0561*), *mut* gene (*SSU0877* and *SSU1797*), *luciferase-like monooxygenase* gene (*SSU0468*), and *ADP ribose pyrophosphatase* gene (*SSU1262*) were investigated following the methodology outlined in the previous work [16].

### Infection experiments

#### Survival assay in C57BL/6 mice infection model

The virulence levels of 32 serotype 5 representative strains, selected from 46 available strains based on their distribution in the phylogenetic tree, were evaluated. For comparison, the highly pathogenic *S. Suis* serotype 2 reference strain P1/7 (ST1) was included [40,41]. Female C57BL/6 mice (6 weeks old) were purchased from SiPeiFu Biotechnology company (Beijing, PR China). The mice were intraperitoneally injected with a standard dose of 2 × 10^7^ CFU for all 32 strains or a low dose of 2 × 10^6^ CFU for strains P1/7, 2020WUSS080, 2020WUSS075, GX169, ID24665, ID48908, YS174, and YS259 in 1 mL of Todd-Hewitt broth (THB). The control group received 1 mL of THB only. Prior to infection, the infection dose of each strain was confirmed by plating serial dilutions of the suspension on THB agar. Based on previous experience [42], each infected group contained ten mice, while the mock-infected group contained five mice. In total, 665 and 175 mice were used in the standard and low-dose infection experiment, respectively. Mortality was recorded to 96 h post-infection and calculated via the Kaplan – Meier method. The experiment was performed independently at least twice for each strain.

#### Pro-inflammatory cytokine production, bacterial loads, and biochemical parameters in peripheral blood of infected C57BL/6 mice

Female C57BL/6 mice (6 weeks old) were intraperitoneally injected with a low dose of 2 × 10^6^ CFU for strains P1/7, 2020WUSS075, 2020WUSS080, GX169, ID24665, and ID48908 in 1 mL THB. The control group received 1 mL of THB only. Based on previous experience [42], each infected group and control group consisted of seven and three mice, respectively. In total, 45 mice were used in the experiment. Peripheral blood was aseptically collected at 9.5 h post-infection. The bacterial colonies in peripheral blood were counted and expressed as CFU/mL. The concentrations of interleukin-6 (IL-6), tumor necrosis factor-α (TNF-α), and monocyte chemoattractant protein-1 (MCP-1) in serum were measured using enzyme-linked immunosorbent assay (ELISA) kits (Thermo Fisher, Carlsbad, CA, USA), following the manufacturer’s recommended protocols. The concentrations of CR, AST, ALT, and LDH in serum were measured using biochemical methods by MAISHA INDUSTRIES company (Yancheng, Jiangsu Province, China).

#### Infection of BV2 microglial cells

The mouse microglia cell line BV2 cells were obtained and prepared according to the description in the previous study [43]. In the infection assay, *S. suis* strains P1/7, 2020WUSS075, 2020WUSS080, GX169, ID24665, and ID48908 (1.5 × 10^6^ CFU/well, 3 wells/strain/each time point) were introduced to BV2 cells at a multiplicity of infection (MOI) of 1 and incubated for 4 and 8 h at 37°C with 5% CO_2_, respectively. Following infection, the cells were washed thrice with phosphate-buffered saline (PBS) and subsequently lysed with TRIzol (0.5 mL per well; Invitrogen) to extract total RNA using an RNA extraction kit (Invitrogen). Pam3CSK4 (TLR1/2 agonist) at a concentration of 50 ng/mL was utilized as a positive control. The expression level of Toll-like receptor 2 (TLR2) in each infected group was analyzed by reverse transcription quantitative PCR (RT-qPCR) according to the methods described in our previous study [40]. The *gapdh* gene (encoding glyceraldehyde 3-phosphate dehydrogenase) was used as a normalizing gene. Non-infected BV2 cells were used as the calibration reference. Each test was performed in duplicate in independent experiments.

### Statistics

Statistical analysis and data visualization for this study were performed using IBM SPSS Statistics version 22 and GraphPad Prism 8. No randomization of samples and strategy to minimize potential confounders was performed. No data were excluded from the data analysis. The normal distribution of the data was firstly determined by the Shapiro–Wilk normality test. Comparisons between two samples with normal distributed data were done with Students *t*-test (analyses of transcription data), while samples without normal distributed data were tested with the Mann–Whitney test (for analyses of cytokine data, bacterial load, and biochemical parameters). The survival curves of different infected groups were compared using the Kaplan – Meier survival analysis with the log-rank test. Data are presented as mean ± SD for normally distributed variables and as median ± IQR for non-normally distributed variables. Differences between infected groups were considered statistically significant at a *p*-value <0.05.

### Ethical approval statement

All mice were bred and maintained in the animal facility of Chinese center for disease control and prevention laboratory animal center with free access to rodent chow and water. The procedures of mouse infection experiment was adhered to the ARRIVE guidelines and approved by the Laboratory Animal Welfare & Ethics Committee of National Institute for Communicable Disease Control and Prevention with permit code 2023–047.

The epidemiological and clinical information of the patient infected with *S. suis* serotype 5 strain GX169 were collected according to the requirement of National Surveillance Program for Human Infection with *Streptococcus suis* (2009 Edition) issued by Chinese Center for Disease Control and Prevention, which was exempted from ethical review and approval by the Laboratory Animal Welfare & Ethics Committee of National Institute for Communicable Disease Control and Prevention. Ethical review and approval of the publicly available data from NCBI and published article (https://doi.org/10.1016/j.micinf.2023.105273) used in the study were not required by the Laboratory Animal Welfare & Ethics Committee of National Institute for Communicable Disease Control and Prevention, because no new collection of human or animal samples were involved in the study.

### Informed consent statement

The written informed consent, adhering to the Helsinki Declaration, has been obtained from the patient infected with *S. suis* serotype 5 strain GX169 to participate in the study and publish the related epidemiological and clinical information for research purposes. Necessary efforts to anonymize the patient were made.

## Results

### Population structure analysis of serotype 5 genomes

In total, 60 distinct sequence types (STs) were identified among the 88 genomes, except for genome YS539, which was non-groupable due to the absence of the *mutS* gene. The predominant STs were ST499 and ST1175, each containing five genomes (Table 1). This finding reveals high heterogeneity within the *S. suis* serotype 5 population.

**Figure 1.**
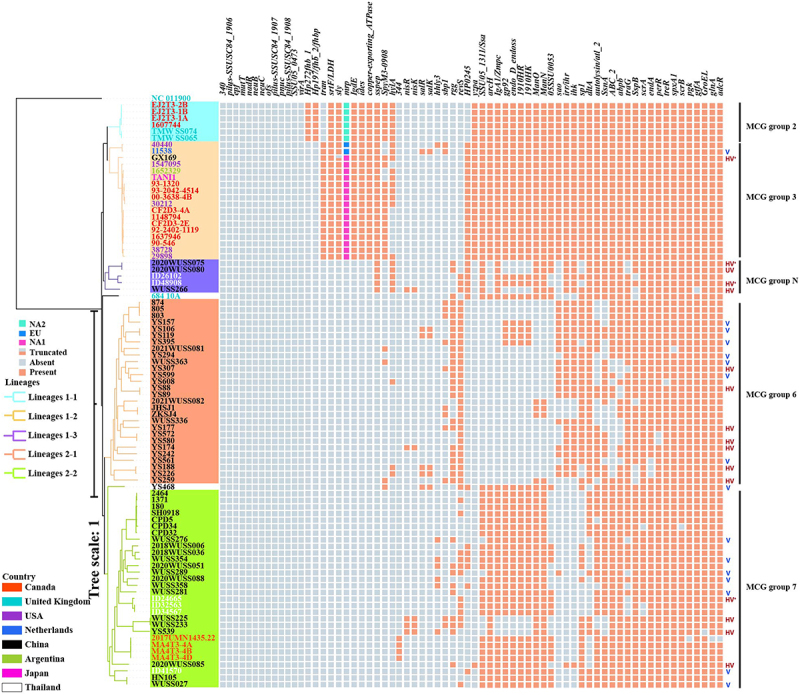
The maximum-likelihood phylogenetic tree of 89 *S. suis* serotype 5 genomes and the distribution of virulence-associated genes in the genomes. The phylogenetic tree was constructed based on the mutant SNPs in the core genomes. The *S. pneumoniae* ATCC 700669 was used as an outgroup to root the tree. The strains were colored based on the geography. The scale is given as the number of substitutions per variable site. UV: ultra-highly virulent, HV: highly virulent, V: virulent.

The serotype 5 genomes were primarily clustered into four MCG groups, including MCG groups 2 (*n* = 6), 3 (*n* = 18), 6 (*n* = 28), and 7–2/3 (*n* = 30). Additionally, seven genomes were MCG non-typable (Table 1 and Figure 1). In the present study, 30 genomes were simultaneously clustered into MCG group 7–2 and MCG group 7–3 with similar confidence values, making it challenging to reliably allocate them into corresponding subgroups. Consequently, the 30 genomes were collectively assigned as MCG group 7–2/3. Conversely, the MCG non-groupable genomes ID26102, ID48908, YS468, 684-10A, 2020WUSS075, 2020WUSS080, and WUSS266 were clustered into MCG group 7–3 with low confidence values of 38.46%, 30.77%, 30.77%, 38.46%, 23.08%, 23.08%, and 30.77%, respectively. Despite ID26102 and ID48908 being assigned as MCG group 7–3 in our previous study [13], the seven genomes were referred to as MCG non-group (group N) in the current study.

MCG groups 2, 3, 6, 7–2/3, and N genomes comprised three, 10, 22, 20, and five STs, respectively. Notably, the MLST non-typeable genome YS539 was clustered into MCG group 7–2/3. MCG typing was relevant to the geographical distribution of serotype 5 genomes. MCG groups 2 and 3 were primarily composed of genomes from Canada, the United States, and the United Kingdom. Conversely, genomes from China were mainly clustered into MCG groups 6 and 7–2/3, while genomes from Thailand were predominantly clustered into MCG group 7–2/3.

To gain further insights into the phylogenetic relationship of serotype 5 genomes, a phylogenetic tree of 89 genomes was constructed based on the distribution of mutant SNPs in the core genome. The serotype 5 genomes were primarily clustered into two lineages, with the MCG non-groupable genome 684-10A forming a distinct branch (Figure 1). Lineage 1 was divided into sub-lineages 1–1 (*n* = 6), 1–2 (*n* = 18), and 1–3 (*n* = 5), corresponding to MCG group 2, 3, and N, respectively. Lineage 2 was primarily divided into sub-lineages 2–1 (*n* = 30) and 2–2 (*n* = 28), comprising MCG groups 6 and 7–2/3, respectively. The MCG non-groupable genome YS468 did not cluster with other Lineage 2 genomes and formed a separate branch.

### Distribution of AR genes and MGEs carrying AR genes in serotype 5 genome

**Figure 2.**
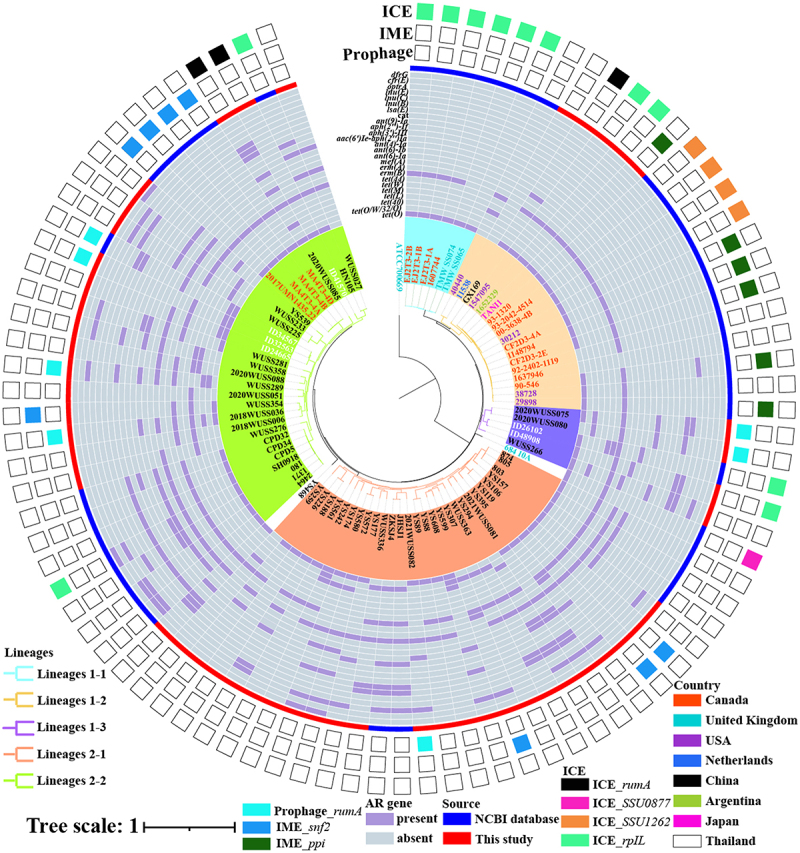
The distribution of AR genes and AR genes-associated MGEs in 89 *S. suis* serotype 5 genomes. Color-filled square boxes on the periphery indicate the presence of AR genes-associated ICEs, IMEs, and prophage integrated into different genes.

A total of 360 AR genes were identified across 87 serotype 5 genomes spanning seven categories, including tetracycline, macrolide-lincosamide-streptogramin (MLS), lincosamide, aminoglycosides, trimethoprim sulfonamides, oxazolidinone, and phenicol. Only two genomes 11538 and 1637946, harbor no known AR genes (Figure 2 and Supplemental Table S1). The serotype 5 genomes contained AR genes conferring resistance to tetracycline and MLS, which were prevalent across serotype 5 genomes. The median number of AR genes in serotype 5 genomes from Lineage 1 and 2 was three and five, respectively. Interestingly, AR genes encoding resistance to trimethoprim sulfonamides, oxazolidinone, and phenicol were exclusively present in genomes originating from China, while AR genes conferring resistance to aminoglycosides were solely identified in genomes from China and Thailand.

Genes coding for resistance to tetracyclines were identified in 85 genomes, belonging to seven distinct types: *tet*(O) (*n* = 67), *tet*(O/W/32/O) (*n* = 12), *tet*(40) (*n* = 11), *tet*(M) (*n* = 9), *tet*(L) (*n* = 4), *tet*(W) (*n* = 2), and *tet*(44) (*n* = 1). Two types of genes responsible for resistance to MLS were identified: the *erm*(B) gene (*n* = 78) and the *erm*(A) gene (*n* = 2). These genes were found in 74 serotype 5 genomes. Additionally, the *mefA* gene, which confers resistance specifically to macrolides, was present in the genome of the WUSS266 strain.

The genes encoding resistance to aminoglycosides were clustered into four categories: i. aminoglycoside O-nucleotidyltransferase *ant(6)- Ia* gene (*n* = 50) and *ant(6)- Ib* gene (*n* = 2), which inactivate streptomycin, and *ant(4”)- Ia* gene (*n* = 11), which inactivates tobramycin and amikacin; ii. aminoglycoside acetyltransferase *aac(6‘) Ie-aph(2’‘) Ia* gene (*n* = 19), which confers resistance to gentamicin and most of the available aminoglycosides; iii. aminoglycoside O-phosphotransferase *aph(3’)-IIIa* gene (*n* = 14), which primarily inactivates kanamycin, and *aph(2””)-If* gene (*n* = 1), which inactivates both kanamycin and gentamicin; iv. aminoglycoside O-nucleotidyltransferase *ant(9)-Ia* gene (*n* = 1), which inactivates spectinomycin.

Eighteen serotype 5 genomes harbored a total of 31 lincosamide resistance genes, including *lsa*(E) (*n* = 13), *lnu*(B) (*n* = 12), *lnu*(C) (*n* = 4), and *lnu*(E) (*n* = 2) genes.

The *optrA* gene, which encodes an ABC F protein and confers resistance to phenicol and oxazolidinone antibiotics, was identified in only 22 genomes from China. Notably, genome SH0918 contained two distinct copies of the *optrA* gene.

The chloramphenicol acetyltransferase gene *cat*, dihydrofolate reductase gene *dfrG*, and 23S ribosomal RNA methyltransferase gene *cfr*(E) gene, which encodes resistance to phenicol, trimethoprim, and phenicol-oxazolidinone-streptogramin-lincosamide were present in 15, four, and two serotype 5 genomes, respectively.

Among serotype 5 genomes, 20 and eight intact ICEs and prophages carrying AR genes were identified, respectively (Figure 2 and Supplemental Table S1). The twelve, four, three, and one intact ICEs were integrated into the *rpIL* gene, ADP-ribose pyrophosphatase gene, *rumA* gene, and *SSU0877* gene, respectively. All prophages were integrated between the *rumA* and *pgmA* genes. Most of the genomes (15/20) harboring the ICEs were clustered into Lineage 1. Furthermore, eight and six IMEs integrated into *snf2* and *ppi* genes were found in serotype 5 genomes, respectively (Figure 2 and Supplemental Table S1). The ICEs, prophage, and IMEs contained 43, 23, and 21 AR genes, respectively (Supplemental Table S1). These AR genes primarily conferred resistance to tetracycline and MLS antibiotics.

The dissemination of the *optrA* gene has led to a rapid increase in phenicol-oxazolidinone (PhO) resistance among *S. suis* isolates [44,45]. This study investigated the genetic elements associated with the spread of the *optrA* gene. Among the 23 *optrA*-positive contigs, 12 were flanked by either *IS1216E* or a truncated version (*ΔIS1216E*) on both ends. In five genomes, the insertion sequence *IS1216E* was present either upstream or downstream of the *optrA* gene (Supplemental Table S1). The *optrA* gene in the genome YS157 was integrated into an IS6 family transposase. In the genomes 2021WUSS082, WUSS281, and WUSS358, the *optrA* genes were located downstream of the transposon Tn554 gene.

### Antimicrobial susceptibility profiles of available strains

Given the importance of β-lactams and glycopeptides in treating *S. suis* infections and the role of the *optrA* gene in antimicrobial resistance, this study investigated the susceptibility of 42 available strains from China to penicillin, cefotaxime, vancomycin, florfenicol, and linezolid (Supplemental Table S2). All strains were susceptible to cefotaxime and vancomycin. However, 28.57% of the strains demonstrated resistance to penicillin with MIC values ranging from 1 to 8 μg/mL. Notably, strain YS294 exhibited high-level resistance to penicillin with an MIC of 8 μg/mL. Among 18 strains harboring the *optrA* gene, the MIC_50_ for florfenicol was 32 μg/mL (ranging from 8 to 64 μg/mL), significantly higher than the MIC_50_ of 2 μg/mL (ranging from <0.5 to 8 μg/mL) in the 24 strains lacking the *optrA* gene. Similarly, the MIC_50_ value for linezolid in the 18 *optrA*-positive strains were 2 μg/mL (ranging from 1 to 4 μg/mL), considerably higher than the MIC_50_ of ≤0.5 μg/mL (ranging from ≤0.5 to 1 μg/mL) in the 24 *optrA*-negative strains (Supplemental Table S3).

### The distribution of known virulence-associated genes in the serotype 5 strains

The distributions of 154 known *S. suis* virulence-associated genes in serotype 5 genomes were investigated. In total, 86 known *S. suis* virulence-associated genes were present in all serotype 5 genomes. A clear difference was observed in the number of absent virulence-associated genes across different MCG groups. The median number of absent virulence-associated genes in MCG group 2, 3, 6, 7–2/3, and N was 24, 25, 46, 40, and 38, respectively. Thirteen virulence associated genes were absent in all serotype 5 genomes, including *epf*, *340*, *SSU05_0473*, *neuB*, *neuC*, *ofs*, *virA*, *mutT*, *pnuc*, *nadR*, and genes encoding accessory pilus subunits (*SSUSC84_1906*, *SSUSC84_1907*, and *SSUSC84_1908*) (Figure 1).

The distributions of some known *S. suis* virulence-associated genes exhibited specificity to MCG groups or lineages. The “classical” virulence-associated genes *mrp* and *sly* were exclusively present in genomes belonging to MCG groups 2 and 3. A premature stop codon was present in *sly* gene of the genome GX169. Notably, a clear distinction in the *mrp* gene genotype was observed between MCG groups 2 and 3. In MCG group 2, the predominant *mrp* genotype was the NA2 subtype, while in MCG group 3, the NA1 subtype was more common. Additionally, the EU subtype was identified in genomes 11,538 and 40,440, both of which belong to MCG group 3.

In addition, *copper-exporting ATPase, srtF*, *ides*, and *IgdE* genes were exclusively found in genomes from MCG groups 2 and 3. The *Hp197* and *Hp272* genes were unique to genomes from MCG group 2, while the *tran* gene was present only in strains from MCG group 3. The *Sspep* gene was identified exclusively in genomes from Lineage 1.

### *cps* loci of serotype 5 genomes

The chromosomal locus of the *cps* gene cluster in the serotype 5 reference genome 11538 (GenBank accession No. BR001003.1) was located between the *orfZ-orfX* region and the *aroA* gene, which belonged to pattern Ia [46]. In the current study, the *cps* gene clusters of 18 serotype 5 genomes were categorized as pattern Ia. All these genomes belonged to Lineages 1–1 and 1–2. In contrast, the *cps* gene clusters of Lineage 1–3, Lineage 2, and the remaining genomes from Lineage 1–1 and Lineage 1–2 were flanked by the *orfZ-orfX* region and the *glf* gene, which belonged to pattern Ib [46] (Table 1).

Genetic heterogeneity was observed within the *cps* gene loci of serotype 5 genomes. The *cps5R* gene encoding aspartate aminotransferase (HG17), along with the *cps5S* (HG18) and *cps5T* (HG19) genes, both encoding hypothetical proteins, were absent in all *cps* gene clusters classified as pattern Ib and in two *cps* gene clusters classified as pattern Ia (strains 1,547,095 and 29,898). Additionally, three types of insertions (HG293+HG294+HG292, HG312+HG313+HG329+HG292, and HG332+HG292) were identified in the 3’ region *cps* gene clusters of pattern Ib and were present in 14, 12, and one strain, respectively.

**Figure 3.**
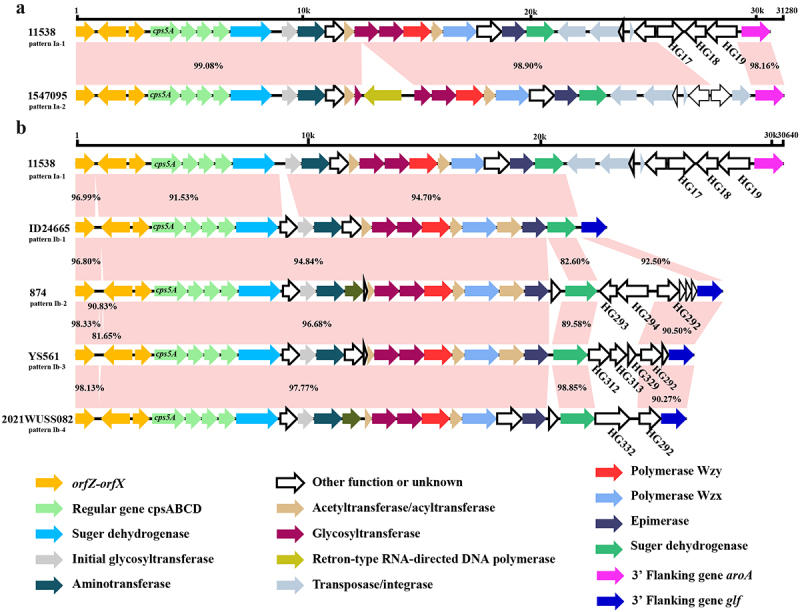
The schematic comparison of the *cps* gene cluster subtype Ia-1 to subtype Ia-2 (a) and subtype Ib (b). Each colored arrow represents the gene whose predicted function is shown in the blow panel. HG17, HG18, HG19, HG292, HG293, HG294, HG312, HG313, HG329, and HG332 genes were indicated. The *orfZ-orfX* genes were located on the 5” side of each locus. The *aroA* or *glf* gene was located on the 3” side of each locus. The identity of different regions was marked by pink shading and corresponding values.

Based on the detection of three homology groups (HG17, HG18, and HG19) and the variable presence of three distinct insertion types, six subtypes were identified among serotype 5 *cps* gene clusters. These subtypes included Ia-1 (*n* = 16), Ia-2 (*n* = 2), Ib-1 (*n* = 44), Ib-2 (*n* = 14), Ib-3 (*n* = 12), and Ib-4 (*n* = 1) (Table 1 and Figure 3).

### The virulence level of serotype 5 strains

This study selected 32 serotype 5 representative strains from 46 available strains to assess their virulence levels. The selection was based on the strain distribution in the core genome phylogenetic tree, encompassing all six available strains from Lineage 1 and 26 out of the 40 available strains from Lineage 2.

Our observations revealed significant variation in virulence levels among *S. suis* serotype 5 strains. By intraperitoneally injecting a standard dose of 2 × 10^7^ CFU per mouse, the survival levels of serotype 5 representative strains were compared to the highly pathogenic serotype 2 strain P1/7. Based on these comparisons, the serotype 5 strains were classified into three groups: ultra-highly virulent (UV), highly virulent (HV), and virulent (V), with 1, 15, and 16 strains in each group, respectively (Table 2). The survival level of mice infected with the UV strain was significantly lower than that of mice infected with the strain P1/7. In contrast, mice infected with HV strains and the strain P1/7 observed a similar survival level. Conversely, mice infected with V strains exhibited a higher survival level than mice infected with the strain P1/7. Among the six strains from Lineage 1, one, four, and one were categorized as UV, HV, and V groups, respectively. Among the 26 strains from Lineage 2, 11 and 15 strains were categorized as HV and V groups, respectively. The strains GX169, ID48908, and ID24665, isolated from patients, were clustered into the HV group, whereas the human strain ID41570 was clustered into the V group (Figure 1). Among the V group, the survival levels of mice infected with strains YS468 and YS561 isolated from healthy pigs were significantly lower than that of mice infected with the human strain ID41570, while the survival levels of mice infected with the remaining 13 strains were similar to that of mice infected with the human strain ID41570 (Table 2).

**Table 2.** The value of mortality and statistical comparison in the survival assay with standard infection dose.

Strains	Survival rate (%) at the corresponding time point							Virulence level	p value		
	6 h	12 h	24 h	36 h	48 h	72 h	96 h		Compared to P1/7 infected group	Compared to ID41570 infected group	Compared to control group
2020WUSS080	30	10	5	5	5	5	5	UV	<0.001	–	<0.01
GX169	95	55	10	0	0	0	0	HV	0.229	–	<0.01
2020WUSS075	65	15	10	0	0	0	0	HV	0.077	–	<0.01
ID48908	90	35	10	10	5	5	5	HV	0.585	–	<0.01
WUSS266	100	35	10	10	5	5	5	HV	0.585	–	<0.01
YS307	100	55	40	30	30	30	30	HV	0.236	–	<0.01
YS88	100	65	30	25	10	10	10	HV	0.076	–	<0.01
YS177	100	50	20	15	15	15	15	HV	0.216	–	<0.01
YS580	100	65	25	20	20	20	20	HV	0.063	–	<0.01
YS174	65	20	0	0	0	0	0	HV	0.123	–	<0.01
YS188	100	25	10	10	5	0	0	HV	0.929	–	<0.01
YS259	80	30	25	15	15	15	15	HV	0.643	–	<0.01
ID24665	95	45	10	5	5	5	5	HV	0.5	–	<0.01
WUSS225	100	55	30	25	20	20	20	HV	0.287	–	<0.01
YS539	100	60	25	15	15	15	10	HV	0.116	–	<0.01
2020WUSS085	100	55	40	25	15	10	10	HV	0.152	–	<0.01
11538	100	80	50	30	25	25	25	V	0.012	0.217	<0.01
YS157	100	100	40	40	25	25	25	V	<0.01	0.384	<0.01
YS106	100	100	35	25	10	10	10	V	<0.01	0.097	<0.01
YS395	100	90	60	50	25	25	25	V	<0.01	0.468	<0.01
YS294	100	95	35	20	15	15	15	V	<0.01	0.094	<0.01
WUSS363	100	100	90	80	30	10	10	V	<0.01	0.894	<0.01
YS599	100	70	35	30	20	20	20	V	0.037	0.059	<0.01
YS561	100	90	30	25	5	5	5	V	<0.01	0.033	<0.01
YS468	100	85	25	20	15	15	15	V	<0.01	0.04	<0.01
WUSS276	100	100	20	20	10	10	10	V	<0.01	0.107	<0.01
WUSS354	100	100	60	30	30	30	30	V	<0.01	0.713	<0.01
WUSS289	100	80	30	30	30	30	30	V	0.027	0.236	<0.01
2020WUSS088	100	100	50	20	20	20	20	V	0.027	0.212	<0.01
WUSS281	100	90	50	40	35	35	35	V	<0.01	0.569	<0.01
WUSS027	100	80	30	30	30	30	30	V	0.027	0.236	<0.01
ID41570	100	90	60	60	40	40	40	V	<0.01	–	<0.01
P1/7	100	20	20	20	10	10	10	HV	–	<0.01	<0.01
Control	100	100	100	100	100	100	100	/	<0.01	<0.01	–

UV: Ultra-highly virulent; HV: Highly virulent; V: Virulent.

The differences in survival levels among the three groups were most pronounced during the early phase of infection. At 6 h post-infection, the survival rate of mice infected with UV strain 2020WUSS080 reached 30%, while both HV and V strain-infected groups maintained a 100% median survival rate at the same time point. At 12 h post-infection, HV and V strain-infected groups exhibited a 50% and 90% median survival rate, respectively. At 48 h post-infection, the survival rate of the V strain-infected mice decreased drastically, reaching a median of 25%. This observation suggests that V strains could induce delayed lethal infection (Table 2).

Among the HV strains, six strains (2020WUSS075, ID48908, ID24665, GX169, YS174, and YS259) induced the death of infected mice before 6 h post-infection, a phenomenon not observed in mice infected with the P1/7 strain. To further evaluate the virulence of these six HV strains and UV strain 2020WUSS080, a lower dose (2 × 10^6^ CFU per mouse) of aforementioned strains was intraperitoneally injected to C57BL/6 mice. The survival rates of mice infected with strains ID48908, ID24665, and GX169 were significantly lower than those infected with the P1/7 strain. Similar survival rates were observed between mice infected with strains 2020WUSS080 and 2020WUSS075, which were significantly lower than those infected with strains ID48908, ID24665, and GX169. However, the survival rates of mice infected with strains YS259 and YS174 remained similar to those infected with the P1/7 strain (Table 3).

**Table 3.** The value of mortality and statistical comparison in the survival assay with low infection dose.

Strains	Survival rate (%) at the corresponding time point							Virulence level	p value		
	6 h	12 h	24 h	36 h	48 h	72 h	96 h		Compared to 2020WUSS080 infected group	Compared to P1/7 infected group	Compared to control group
2020WUSS080	100	30	15	5	5	5	5	UV	–	<0.01	<0.01
2020WUSS075	100	50	40	10	10	10	10	HV*+*	0.222	<0.01	<0.01
GX169	100	70	33.3	16.7	10	10	10	HV*+*	<0.01	<0.01	<0.01
ID48908	100	80	30	0	0	0	0	HV*+*	0.029	<0.01	<0.01
YS174	100	65	50	50	50	50	50	HV	<0.01	0.178	<0.01
YS259	100	100	60	50	50	50	50	HV	<0.01	0.534	0.012
ID24665	100	90	20	20	20	20	20	HV*+*	<0.01	<0.01	<0.01
P1/7	100	80	70	56.7	56.7	56.7	56.7	HV	<0.01	–	<0.01
Control	100	100	100	100	100	100	100	/	<0.01	<0.01	–

UV: ultra-highly virulent; HV^+^: Highly virulent plus; HV: Highly virulent.

The differences in survival levels among the infected groups also primarily occurred during the early phase of infection using a low dose. At 12 h post-infection, mice infected with strains 2020WUSS080 and 2020WUSS075 had 30% and 50% survival rates, respectively. In contrast, at the same time point, mice infected with strains ID24665, ID48908, and GX169 had survival rates of 90%, 80%, and 70%, respectively. However, at 36 h post-infection, the survival rates of mice infected with strains ID24665, ID48908, and GX169 decreased dramatically, reaching 20%, 0%, and 16.7%, respectively, similar to those of mice infected with strains 2020WUSS080 and 2020WUSS075. Conversely, mice infected with strain P1/7 had 100% and 70% survival rates at 12 h and 36 h post-infection, respectively (Table 3).

Our observation indicated that the virulence of serotype 5 strains 2020WUSS075, ID24665, ID48908, and GX169 was significantly higher than strain P1/7 using a low dose. In this study, strains 2020WUSS075, ID24665, ID48908, and GX169 were re-classified into the highly virulent plus (HV^+^) group (Figure 1).

### The mechanism related to the pathogenicity increase of HV^+^ strains

#### Bacterial loads, pro-inflammatory cytokines production, and biochemical parameters in peripheral blood of infected mice

The bacterial loads, pro-inflammatory cytokine levels, and biochemical parameters in the peripheral blood of mice intraperitoneally infected with serotype 5 UV strain, HV^+^ strains, and serotype 2 strain P1/7 were compared using a low dose. As the mice infected with the UV strain 2020WUSS080 were dying at 9.5 h post-infection, the peripheral blood of the infected mice was collected at that time point.

**Figure 4.**
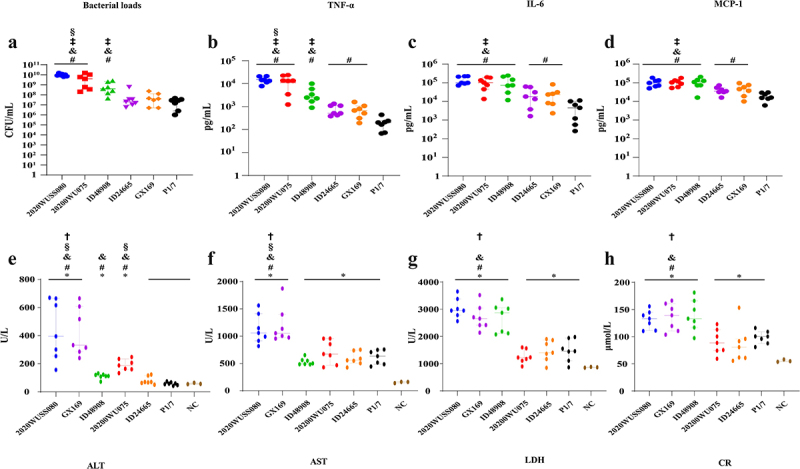
The bacterial loads in peripheral blood (a), concentrations of pro-inflammatory cytokines IL-6 (b), TNF-α (c), MCP-1(d), and biochemical parameters ALT (e), AST (f), LDH (g), and CR (h) in serum of C57BL/6 mice intraperitoneally infected with 2 × 10^6^ CFU of serotype 5 strains 2020WUSS080, 2020WUSS075, ID48908, GX169, ID24665, and serotype 2 strain P1/7 at 9.5 h post-infection. Bacterial count, pro-inflammatory cytokines value, and biochemical parameters value of individuals, including median with interquartile ranges, were presented. Statistical differences in bacterial count, pro-inflammatory cytokines, and biochemical parameter values among infected groups were determined using Wilcoxon’s rank sum test. *p*<0.05 was considered significant.

The highest bacterial load was observed in mice infected with UV strain 2020WUSS080 and HV^+^ strain 2020WUSS075, both exceeding a median value of 10^9^ CFU/mL. In contrast, mice infected with HV^+^ strain ID48908 exhibited significantly lower bacterial loads, which were still higher than those observed in mice infected with strains GX169, ID24665, and P1/7 (Figure 4(a)).

*: significantly higher than those of control groups.

#: significantly higher than those of strain P1/7 infected groups.

&;: significantly higher than those of HV^+^ strain ID24665 infected group.

§: significantly higher than those of HV^+^ strain ID48908 infected group.

†: significantly higher than those of HV^+^ strain 2020WUSS075 infected group.

‡: significantly higher than those of HV^+^ strain GX169 infected group.

The mice infected with UV strain 2020WUSS080 and HV^+^ strain 2020WUSS075 produced the highest serum levels of TNF-α. In comparison, strain ID48908 elicited higher TNF-α levels than strains GX169, ID24665, and P1/7. The TNF-α level was similar between the mice infected with strains GX169 and ID24665, while they were significantly higher than that of mice infected with strain P1/7 (Figure 4(b)).

The levels of IL-6 and MCP-1 in the serum of mice infected with UV strain 2020WUSS080, HV^+^ strains 2020WUSS075 and ID48909 were similar, which were significantly higher than those of mice infected with strains GX169, ID24665, and P1/7. Mice infected with HV^+^ strains GX169 and ID24665 exhibited significantly higher IL-6 and MCP-1 levels in serum compared to strain P1/7-infected mice, while no significant difference was observed between GX169 and ID24665-infected mice (Figure 4(c,d), Supplemental Table S4).

Serum biomarkers associated with tissue integrity or function, including ALT, AST, LDH, and CR were measured. ALT is primarily present in the cytoplasm of hepatocytes. Myocardial, hepatocellular, and muscle damages are the main sources of serum AST. LDH and CR primarily reflected the myocardial and kidney damage, respectively. The UV strain 2020WUSS080 and the HV^+^ strain GX169 induced the highest serum levels of ALT and AST among the six infected groups. In contrast, the highest levels of LDH and CR were observed in the groups infected with UV strain 2020WUSS080 and HV^+^ strains GX169 and ID48908. Compared to the ALT serum level in the control group, no significant upregulation was observed in the ID24665 and P1/7-infected groups, which exhibited significantly lower levels than those infected with HV^+^ strains 2020WUSS075 and ID48908. In addition, the ALT serum level in HV^+^ strain 2020WUSS075-infected group was significantly higher than that of group infected with HV^+^ strain ID48908. Similar serum AST levels were observed in the 2020WUSS075, ID48908, ID24665, and P1/7-infected groups, all significantly higher than those of the control group. Compared to the control group, significant upregulation of serum LDH and CR levels was also found in the HV^+^ strains 2020WUSS075 and ID24665-infected groups, which were similar to those of the P1/7 infected group (Figure 4(e–h), Supplemental Table S4).

#### TLR2 mRNA expression levels in microglial cells

**Figure 5.**
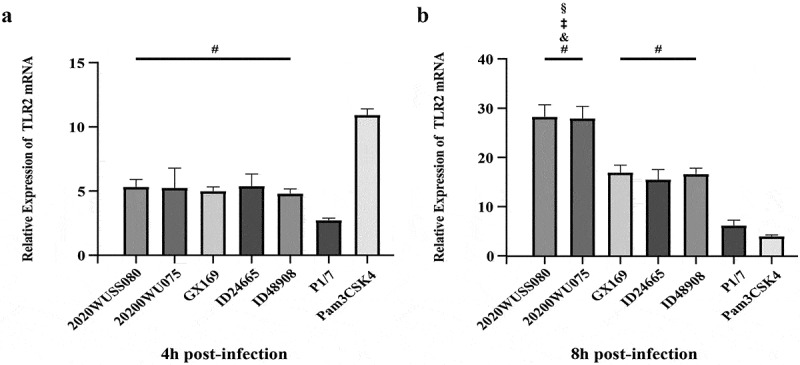
The transcription levels of TLR2 genes in BV2 cells infected with serotype 5 strains 2020WUSS080, 2020WUSS075, ID48908, GX169, ID24665, and serotype 2 strain P1/7 at 4 h (a) and 8 h (b) post-infection. the transcription levels of infected mouse microglia cell line BV2 cells at MOI of 1 were calculated after normalizing cycle thresholds against the “housekeeping” gene *gapdh* using the 2^−ΔΔCt^ method. All data were presented as mean ± standard deviation. Statistical analyses of the data were performed using the Student unpaired *t* test. *p*<0.05 was considered as significant.

TLR2 is critical in the lethal inflammatory response induced by *S. suis* strain infection. In the present study, the TLR2 expression levels of the mouse microglia cell line BV2 cells infected with UV strain, HV^+^ strains, and P1/7 were compared at 4 h and 8 h post-infection. The expression levels of TLR2 were upregulated at 4 h post-infection and further increased at 8 h post-infection in all infected groups (Figure 5). At 4 h post-infection, the TLR2 expression levels in groups infected with UV and HV^+^ strains were significantly higher than that of the group infected with strain P1/7. However, no significant differences in TLR2 expression levels were observed among the groups infected with serotype 5 strains (Figure 5(a)). At 8 h post-infection, similar TLR2 expression levels were observed between UV strain 2020WUSS080 and HV^+^ strain 2020WUSS075 infected groups, which were significantly higher than those of the groups infected with HV^+^ stains ID24665, ID48908, and GX169. The TLR2 expression levels in the groups infected with HV^+^ stains ID24665, ID48908, and GX169 were similar and significantly higher than the level observed in the group infected with strain P1/7 (Figure 5(b)).

#: significantly higher than those of strain P1/7 infected groups.

&;: significantly higher than those of HV^+^ strain ID24665 infected group.

‡: significantly higher than those of HV^+^ strain GX169 infected group.

§: significantly higher than those of HV^+^ strain ID48908 infected group.

## Discussion

Worldwide, serotype 2 is the primary zoonotic serotype of *S. suis* [2]. In recent years, serotype 14 has also been frequently isolated from human *S. suis* infections [42,47,48]. Over the last decade, serotype 5 has emerged as the third most common serotype in human *S. suis* cases [7]. In our previous study, genomes of eight *S. suis* serotype 5 strains were analyzed, and pathogenic potential was revealed [13]. Considering the substantial threat posed by *S. suis* serotype 5 strains to public health and the pig industry globally, there is an urgent need to evaluate the epidemiology and pathogenicity of *S. suis* serotype 5 population.

The phylogenetic population of serotype 5 genomes comprised two distinct lineages. Lineage 1 consisted of MCG groups 2, 3, and N genomes, while Lineage 2 comprised MCG groups 6 and 7–2/3 genomes. Notably, serotype 5 strains isolated from patients were clustered into MCG groups 3, 7–2/3, and N. Most human *S. suis* infections were caused by serotype 2 and 14 strains belonging to MCG group 1, which was previously considered the sole zoonotic group [36]. Over the past decade, human infections with *S. suis* serotypes 1, 4, 5, 7, 9, 16, 21, 24, and 31 have been reported in Thailand, Vietnam, and China [13–19,21,22]. Furthermore, the serotype 4 and 7 strains from human cases were highly virulent in zebrafish and mice [16,39]. The emerging zoonotic serotype 1, 4, 7, 24, and 31 strains were also clustered into MCG groups 3, 7, and N [13–16], indicating the continuous emergence and evolution of zoonotic *S. suis* MCG groups. Recently, ST1656 strains, identified as the culprit of the first *S. suis* outbreak associated with consuming a raw pork dish in Thailand [49], were clustered into MCG group 4. Similarly, a novel *S. suis* ST strain isolated from the first human *S. suis* case linked to the consumption of a raw pork dish in Korea [50] was clustered into MCG group 3. The fact that emerging human strains of different serotypes belonged to the same ST suggests that the *cps* gene locus was exchanged between strains of different serotypes, including ST221 (for both serotype 24 and 31 strains) and ST373 (for both serotype 5 and 7 strains). Previous studies have shown that *cps* switching increases the zoonotic potential of *S. suis* strains [51]. The gastrointestinal tract is a major entry point for *S. suis* infections in Thailand. *In vitro* studies have demonstrated that CPS plays differentiated roles in modulating the interaction of *S. suis* with various intestinal epithelial cells [51]. It should be emphasized that the *cps* switch may play important roles in the continuous emergence of the zoonotic *S. suis* strains.

The different chromosomal loci of the *cps* gene clusters revealed the different evolutionary histories of corresponding strains. Pattern Ia *cps* gene cluster was exclusively found among genomes from Lineage 1, while all *cps* gene clusters of genomes from Lineage 2 were classified as pattern Ib. Furthermore, obvious variations in arrangement of *cps* gene cluster were observed between two lineages. The subtypes of serotype 5 *cps* gene cluster Ib-2, Ib-3, and Ib-4 were exclusively identified in genomes from China of Lineage 2 based on the variable presence of HG292, HG293, HG294, HG312, HG313, HG329, and HG332 identified in *S. suis* NCLs strains from China [52]. It is possible that the horizontal gene transfer of NCL-specific HGs occurred in genomes of serotype 5 Lineage 2, suggesting that distinct evolutionary events happened between the genomes of two lineages.

The number of AR genes harbored in serotype 5 genomes from Lineage 2 was significantly higher than those from Lineage 1. The rise of antimicrobial resistance to oxazolidinones, including linezolid, tedizolid, and contezolid, due to the spread of the *optrA* gene, is emerging as a potential threat to public health [45]. All serotype 5 genomes containing the *optrA* gene were isolated in China and belonged to Lineage 2. In the study, the presence of *optrA* gene significantly increased the resistance of strains to linezolid and florfenicol. Three types of transposase genes were found in the flanking region of *optrA* genes. Furthermore, the *optrA* gene identified in genome SH0918 was integrated into plasmid pSH0918 [45]. Additionally, the *optrA* gene harbored in genome WUSS289 was incorporated into prophage. The MGEs may facilitate the spread of the *optrA* gene in *S. suis*, presenting an emerging threat to public health in China. Compared to the genomes of Lineage 2, the MGEs carrying the AR genes encoding tetracycline and MLS resistance were widespread in genomes of Lineage 1, indicating the dissemination mechanism of corresponding AR genes was different between the two lineages. Considering the genomes from Lineage 2 harboring more AR genes, further studies are needed to investigate their dissemination mechanisms. Susceptibility testing confirmed that cefotaxime and vancomycin are effective options for treating *Streptococcus suis* infections.

The genomes from the two lineages exhibited significant differences in evolutionary and genomic characteristics. We propose that the two lineages evolved in parallel, with genomes from Lineage 2 undergoing high rates of recombination events and rapid population expansion. Considering the substantial recombination in *S. suis* and to accurately classify *S. suis* strains into corresponding subpopulations, our MCG typing system removed recombinant regions instead of entire genes [36]. Compared to genomes from MCG groups 2, 3, and 6, genomes from MCG group 7 had a significantly more extensive recombination history [36]. The recombinant SNP sites may not have been thoroughly identified, and some were classified as MCG 7 sub-group-specific SNPs in our MCG scheme. It is possible that some of these SNPs underwent reverse or parallel mutations in the serotype 5 genomes from MCG group 7, adversely affecting the assignment of MCG 7 sub-groups. To precisely assign strains to corresponding subpopulations and reflect their phylogenetic relationships, novel strategies to exclude recombinant SNPs are necessary.

Significant heterogeneity in virulence was observed among serotype 5 strains, clustered into the UV, HV^+^, HV, and V groups. Virulence levels progressively decreased from the UV group to HV^+^, HV, and V groups. In this study, one strain from MCG group N was clustered into the UV group, one from MCG group 3, one from MCG group 7–2/3, and two from MCG group N were clustered into the HV^+^ group. The virulence levels of strains in the UV and HV^+^ groups were significantly higher than that of the highly pathogenic strain P1/7, as characterized by the significantly higher mortality of infected mice during the early phase of infection. Based on our observations, the stronger the pathogenicity of *S. suis* strains toward C57BL/6 mice, the greater its ability to induce severe clinical symptoms in humans [40,53,54]. These findings suggest that UV and HV^+^ strains, which demonstrated higher pathogenicity in C57BL/6 mice, may pose a higher risk of human infection. Notably, both UV strain 2020WUSS080 and HV^+^ strain 2020WUSS075 were isolated from healthy pigs. Furthermore, 11 strains isolated from healthy pigs were clustered into the HV group, which exhibited a virulence level similar to that of the highly pathogenic strain P1/7. In the present study, strain ID41570 isolated from a sepsis patient was clustered into V group. Among the V group, the virulence level of 13 strains isolated from healthy pigs was higher than or similar to strain ID41570. *S. suis* strains isolated from healthy pigs were highly virulent and were a source of infection to humans [39,55,56]. Our finding indicates that the serotype 5 strains isolated from healthy pigs possess significant zoonotic potential. These observations highlight the significant public health threat posed by *S. suis* serotype 5 strains and the necessity of continuous surveillance.

In our previous study, the capacity to overproduce TNF-α in serum and promptly establish infection played critical roles in the sudden death of infected mice during the early phase of infection [42]. The current study found that serotype 5 UV strain possessed a higher capacity to resist host immune clearance, replicate, disseminate, and establish infection, subsequently triggering the production of excessive TNF-α in the early stages of infection. The significant difference in capacities to induce organ damage of infected mice were observed among UV strain and HV^+^ strains, which was not entirely due to the differences in bacterial loads. Furthermore, the relatively higher AST/ALT ratio and the significantly elevated LDH levels indicated that the elevated serum AST in mice infected with strain ID24665 may primarily result from myocardial damage, while the elevated serum AST in other infected groups primarily originated from liver damage. Our data indicated that the mechanisms of *S. suis* serotype 5 UV and HV^+^ strains to induce organ damage are different and need further investigations.

The role of TLR2 in the inflammatory response induced by *S. suis* strains is well established [57]. To further elucidate the mechanism underlying the differences in pro-inflammatory cytokine production *in vivo* between strains, the expression levels of TLR2 in BV2 cells infected with different strains *in vitro* were compared. The capacity to activate TLR2 expression was significantly higher in serotype 5 UV and HV^+^ strains compared to strain P1/7. Moreover, the expression levels of TLR2 in cells infected with UV strain 2020WUSS080 and HV^+^ strain 2020WUSS075 were significantly higher than those infected with HV^+^ strains ID48908, ID24665, and GX169. These findings suggest that TLR2 partially contributes to the excessive production of pro-inflammatory cytokines induced by serotype 5 UV and HV^+^ strain infections and is responsible for the difference in pro-inflammatory cytokine levels among HV^+^ strains. However, further research is necessary to determine whether other receptors or pathways are also involved in the exacerbated inflammatory response elicited by these strains.

Among the serotype 5 UV and HV^+^ strains, no significant difference in mortality was observed between the standard dose and corresponding low-dose infected groups at 96 h post-infection. Conversely, an apparent disparity in mortality was noted between mice infected with standard and corresponding low doses of strain P1/7 at 96 h post-infection. This suggests that lethal infections caused by serotype 5 UV and HV^+^ strains are dose-independent but time-dependent. Moreover, the lethal doses of serotype 5 UV and HV^+^ strains were markedly lower compared to that of strain P1/7, attributable to their superior capacities for establishing infection and activating inflammatory pathways.

Strains of *S. suis* from MCG group 1 possessed the highest numbers of known virulence-associated genes. In contrast, serotype 5 UV and HV^+^ strains from MCG group 3, 7, and N carried significantly fewer known virulence-associated genes [36]. Moreover, virulence-associated genes such as *epf*, *sly*, *ofs*, *nisK*, *nisR*, *salK*, *salR*, *nadR*, *neuB*, and *neuC*, which are preferentially present in highly pathogenic serotype 2 strains of *S. suis* [58–60], were absent from all serotype 5 UV and HV^+^ strains. It is reasonable to hypothesize that the pathogenesis of serotype 5 UV and HV^+^ strains differed from that of MCG group 1 strains. The virulence level of serotype 5 strains cannot be accurately assessed solely by the presence of known virulence-associated genes, which were primarily identified from MCG group 1 strains. Currently, limited information is available regarding the genes associated with the virulence level of non-MCG group 1 strains. To establish effective control strategies, further studies on the pathogenesis of non-MCG group 1 strains are critically needed.

In the conclusion, the genomic heterogeneity and varying virulence levels within the *S. suis* serotype 5 population were revealed. The pathogenic features of serotype 5 UV and HV^+^ strains differed markedly from those of the highly pathogenic serotype 2 strain P1/7. These findings deepen our understanding of *S. suis* serotype 5 epidemiology and pathogenicity, offering valuable insights into its zoonotic potential and providing important information for controlling infections associated with these strains.

## Supplementary Material

Supplemental Table 4.docx

Supplemental Table 3.docx

Supplemental Table 2.docx

Supplemental Table 1.docx

## Data Availability

The sequence of the genome sequenced in the study was deposited in the GenBank under accession numbers SAMN33923801-SAMN33923842. The data that support the findings of this study and the supplementary materials are openly available in Figshare at https://doi.org/10.6084/m9.figshare.28060139.

## References

[1] Segura M, Zheng H, de Greeff A, et al. Latest developments on streptococcus suis: an emerging zoonotic pathogen: part 1. Future Microbiol. 2014;9(4):441–21. doi: 10.2217/fmb.14.14 24810343

[2] Huong VT, Ha N, Huy NT, et al. Epidemiology, clinical manifestations, and outcomes of streptococcus suis infection in humans. Emerg Infect Dis. 2014;20(7):1105–1114. doi: 10.3201/eid2007.131594 24959701 PMC4073838

[3] Goyette-Desjardins G, Auger JP, Xu J, et al. Streptococcus suis, an important pig pathogen and emerging zoonotic agent-an update on the worldwide distribution based on serotyping and sequence typing. Emerg Microbes Infect. 2014;3(6):1–20. doi: 10.1038/emi.2014.45 PMC4078792 26038745

[4] Kerdsin A, Segura M, Fittipaldi N, et al. Sociocultural factors influencing human streptococcus suis Disease in Southeast Asia. Foods. 2022;11(9):1190. doi: 10.3390/foods11091190 35563913 PMC9102869

[5] Haas B, Grenier D. Understanding the virulence of streptococcus suis: a veterinary, medical, and economic challenge. Medecine et Mal infectieuses. 2018;48(3):159–166. doi: 10.1016/j.medmal.2017.10.001 29122409

[6] Kerdsin A. Human streptococcus suis infections in Thailand: epidemiology, clinical features, genotypes, and susceptibility. Trop Med Infect Dis. 2022;7(11):359. doi: 10.3390/tropicalmed7110359 36355901 PMC9695567

[7] Segura M, Aragon V, Brockmeier SL, et al. Update on streptococcus suis research and prevention in the era of antimicrobial restriction: 4th international workshop on S. suis. Pathogens. 2020;9(5):374. doi: 10.3390/pathogens9050374 32422856 PMC7281350

[8] Guntala R, Khamai L, Srisai N, et al. Contamination of streptococcus suis and S. suis serotype 2 in raw pork and edible pig organs: a public health concern in Chiang Mai, Thailand. Foods. 2024;13(13):2119. doi: 10.3390/foods13132119 38998625 PMC11241745

[9] Dutkiewicz J, Zajac V, Sroka J, et al. Streptococcus suis: a re-emerging pathogen associated with occupational exposure to pigs or pork products. Part II - Pathogenesis. Ann Agric Environ Med. 2018;25(1):186–203. doi: 10.26444/aaem/85651 29575852

[10] Okura M, Osaki M, Nomoto R, et al. Current taxonomical situation of streptococcus suis. Pathogens. 2016;5(3):45. doi: 10.3390/pathogens5030045 27348006 PMC5039425

[11] Tien le HT, Nishibori T, Nishitani Y, et al. Reappraisal of the taxonomy of streptococcus suis serotypes 20, 22, 26, and 33 based on DNA-DNA homology and sodA and recN phylogenies. Vet Microbiol. 2013;162(2–4):842–849. doi: 10.1016/j.vetmic.2012.11.001 23245487

[12] Kralova N, Fittipaldi N, Zouharova M, et al. Streptococcus suis strains with novel and previously undescribed capsular loci circulate in Europe. Vet Microbiol. 2024;298:110265. doi: 10.1016/j.vetmic.2024.110265 39340873

[13] Kerdsin A, Hatrongjit R, Wongsurawat T, et al. Comparative genome analysis of streptococcus suis serotype 5 strains from humans and pigs revealed pathogenic potential of virulent, antimicrobial resistance, and genetic relationship. Microbes Infect. 2023;27(1):105273. doi: 10.1016/j.micinf.2023.105273 38070594

[14] Hatrongjit R, Fittipaldi N, Jenjaroenpun P, et al. Genomic comparison of two streptococcus suis serotype 1 strains recovered from porcine and human disease cases. Sci Rep. 2023;13(1):5380. doi: 10.1038/s41598-023-32724-z 37009816 PMC10068604

[15] Hatrongjit R, Boueroy P, Jenjaroenpun P, et al. Genomic characterization and virulence of streptococcus suis serotype 4 clonal complex 94 recovered from human and swine samples. PLOS ONE. 2023;18(7):e0288840. doi: 10.1371/journal.pone.0288840 37498866 PMC10374156

[16] Liang P, Wang M, Gottschalk M, et al. Genomic and pathogenic investigations of streptococcus suis serotype 7 population derived from a human patient and pigs. Emerg Microbes Infect. 2021;10(1):1960–1974. doi: 10.1080/22221751.2021.1988725 34635002 PMC8525962

[17] Kerdsin A, Hatrongjit R, Wongsurawat T, et al. Genomic characterization of streptococcus suis serotype 24 clonal complex 221/234 from human patients. Front Microbiol. 2021;12:812436. doi: 10.3389/fmicb.2021.812436 35003043 PMC8733411

[18] Kerdsin A, Hatrongjit R, Gottschalk M, et al. Emergence of streptococcus suis serotype 9 infection in humans. J Microbiol Immunol Infect. 2017;50(4):545–546. doi: 10.1016/j.jmii.2015.06.011 26362754

[19] Hatrongjit R, Kerdsin A, Gottschalk M, et al. First human case report of sepsis due to infection with streptococcus suis serotype 31 in Thailand. BMC Infect Dis. 2015;15(1):392. doi: 10.1186/s12879-015-1136-0 26420029 PMC4588491

[20] Kerdsin A, Oishi K, Sripakdee S, et al. Clonal dissemination of human isolates of streptococcus suis serotype 14 in Thailand. J Med Microbiol. 2009;58(Pt 11):1508–1513. doi: 10.1099/jmm.0.013656-0 19661209

[21] Nghia HD, Hoa NT, Linh le D, et al. Human case of streptococcus suis serotype 16 infection. Emerg Infect Dis. 2008;14(1):155–157. doi: 10.3201/eid1401.070534 18258097 PMC2600150

[22] Callejo R, Prieto M, Salamone F, et al. Atypical streptococcus suis in man, Argentina, 2013. Emerg Infect Dis. 2014;20(3):500–502. doi: 10.3201/eid2003.131148 24565286 PMC3944841

[23] Cloutier G, D’Allaire S, Martinez G, et al. Epidemiology of streptococcus suis serotype 5 infection in a pig herd with and without clinical disease. Vet Microbiol. 2003;97(1–2):135–151. doi: 10.1016/j.vetmic.2003.09.018 14637045

[24] Gottschalk M, Lacouture S, Bonifait L, et al. Characterization of streptococcus suis isolates recovered between 2008 and 2011 from diseased pigs in Quebec, Canada. Vet Microbiol. 2013;162(2–4):819–825. doi: 10.1016/j.vetmic.2012.10.028 23177911

[25] Bojarska A, Janas K, Pejsak Z, et al. Diversity of serotypes and new cps loci variants among streptococcus suis isolates from pigs in Poland and Belarus. Vet Microbiol. 2020;240:108534. doi: 10.1016/j.vetmic.2019.108534 31902504

[26] Prufer TL, Rohde J, Verspohl J, et al. Molecular typing of streptococcus suis strains isolated from diseased and healthy pigs between 1996–2016. PLOS ONE. 2019;14(1):e0210801. doi: 10.1371/journal.pone.0210801 30653570 PMC6336254

[27] Murray GGR, Hossain A, Miller EL, et al. The emergence and diversification of a zoonotic pathogen from within the microbiota of intensively farmed pigs. Proc Natl Acad Sci USA. 2023;120(47):e2307773120. doi: 10.1073/pnas.2307773120 37963246 PMC10666105

[28] Kerdsin A, Dejsirilert S, Sawanpanyalert P, et al. Sepsis and spontaneous bacterial peritonitis in Thailand. Lancet. 2011;378(9794):960. doi: 10.1016/S0140-6736(11)60923-9 21890062

[29] Gomez E, Kennedy CC, Gottschalk M, et al. Streptococcus suis-related prosthetic joint infection and streptococcal toxic shock-like syndrome in a pig farmer in the United States. J Clin Microbiol. 2014;52(6):2254–2258. doi: 10.1128/JCM.02934-13 24719433 PMC4042794

[30] Callejo R, Zheng H, Du P, et al. Streptococcus suis serotype 2 strains isolated in Argentina (South America) are different from those recovered in North America and present a higher risk for humans. JMM Case Rep. 2016;3(5):e005066. doi: 10.1099/jmmcr.0.005066 28348788 PMC5343146

[31] Gustavsson C, Rasmussen M. Septic arthritis caused by streptococcus suis serotype 5 in pig farmer. Emerg Infect Dis. 2014;20(3):489–491. doi: 10.3201/eid2003.130535 24565084 PMC3944842

[32] Taniyama D, Sakurai M, Sakai T, et al. Human case of bacteremia due to streptococcus suis serotype 5 in Japan: the first report and literature review. IDCases. 2016;6:36–38. doi: 10.1016/j.idcr.2016.09.011 27689023 PMC5040640

[33] Chen LX, Dong GY, Wen LQ. Analysis o fthe pathogenic characteristics of 36 isolates of streptococcus suis from human-originin Fujian Province. Chin J Zoonoses. 2024;40(2):161–165,170. doi: 10.3969/j.issn.1002-2694.2024.00.026

[34] Wen LQ, Huo LH, Ying HM, et al. Etiological diagnosis, typing, and detection of related genes in isolates from a case of streptococcus suis type 5 infection. Chin J Zoonoses. 2021;37(4):346–350,361. doi: 10.3969/j.issn.1002-2694.2021.00.034

[35] Chatellier S, Harel J, Zhang Y, et al. Phylogenetic diversity of streptococcus suis strains of various serotypes as revealed by 16S rRNA gene sequence comparison. Int J Systematic Bacteriol. 1998;48(Pt 2):581–589. doi: 10.1099/00207713-48-2-581 9731300

[36] Chen C, Zhang W, Zheng H, et al. Minimum core genome sequence typing of bacterial pathogens: a unified approach for clinical and public health microbiology. J Clin Microbiol. 2013;51(8):2582–2591. doi: 10.1128/JCM.00535-13 23720795 PMC3719615

[37] Seemann T. Prokka: rapid prokaryotic genome annotation. Bioinformatics. 2014;30(14):2068–2069. doi: 10.1093/bioinformatics/btu153 24642063

[38] Hu Y, Yang X, Qin J, et al. Metagenome-wide analysis of antibiotic resistance genes in a large cohort of human gut microbiota. Nat Commun. 2013;4(1):2151. doi: 10.1038/ncomms3151 23877117

[39] Zhu J, Wang J, Kang W, et al. Streptococcus suis serotype 4: a population with the potential pathogenicity in humans and pigs. Emerg Microbes Infect. 2024;13(1):2352435. doi: 10.1080/22221751.2024.2352435 38703011 PMC11097711

[40] Lachance C, Gottschalk M, Gerber PP, et al. Exacerbated type II interferon response drives hypervirulence and toxic shock by an emergent epidemic strain of streptococcus suis. Infect Immun. 2013;81(6):1928–1939. doi: 10.1128/IAI.01317-12 23509145 PMC3676015

[41] Holden MT, Hauser H, Sanders M, et al. Rapid evolution of virulence and drug resistance in the emerging zoonotic pathogen Streptococcus suis. PLOS ONE. 2009;4(7):e6072. doi: 10.1371/journal.pone.0006072 19603075 PMC2705793

[42] Kang W, Wang M, Yi X, et al. Investigation of genomic and pathogenicity characteristics of streptococcus suis ST1 human strains from Guangxi Zhuang Autonomous region (GX) between 2005 and 2020 in China. Emerg Microbes Infect. 2024;13(1):2339946. doi: 10.1080/22221751.2024.2339946 38578304 PMC11034456

[43] Wang J, Liang P, Sun H, et al. Comparative transcriptomic analysis reveal genes involved in the pathogenicity increase of streptococcus suis epidemic strains. Virulence. 2022;13(1):1455–1470. doi: 10.1080/21505594.2022.2116160 36031944 PMC9423846

[44] Wang CZ, Wang MG, Chu YF, et al. Antibiotic resistance patterns and molecular characterization of streptococcus suis isolates from swine and humans in China. Microbiol Spectr. 2023;11(3). doi: 10.1128/spectrum.00309-23 PMC10269843 37154736

[45] Dai XY, Sun JJ, Zhu BQ, et al. Various Mobile genetic elements involved in the dissemination of the phenicol-oxazolidinone resistance gene optrA in the zoonotic pathogen streptococcus suis: a nonignorable risk to public health. Microbiol Spectr. 2023;11(3):ARTN e04875–22. doi: 10.1128/spectrum.04875-22 PMC10269897 37070987

[46] Okura M, Takamatsu D, Maruyama F, et al. Genetic analysis of capsular polysaccharide synthesis gene clusters from all serotypes of streptococcus suis: potential mechanisms for generation of capsular variation. Appl Environ Microbiol. 2013;79(8):2796–2806. doi: 10.1128/AEM.03742-12 23416996 PMC3623174

[47] Wang M, Du P, Wang J, et al. Genomic epidemiology of streptococcus suis sequence type 7 sporadic infections in the Guangxi Zhuang Autonomous region of China. Pathogens. 2019;8(4):187. doi: 10.3390/pathogens8040187 31614790 PMC6963630

[48] Ji L, Chen Z, Li F, et al. Epidemiological and genomic analyses of human isolates of streptococcus suis between 2005 and 2021 in Shenzhen, China. Front Microbiol. 2023;14:1118056. doi: 10.3389/fmicb.2023.1118056 37113229 PMC10126776

[49] Brizuela J, Kajeekul R, Roodsant TJ, et al. Streptococcus suis outbreak caused by an emerging zoonotic strain with acquired multi- drug resistance in Thailand. Microb Genomics. 2023;9(2):ARTN 000952. doi: 10.1099/mgen.0.000952 PMC9997742 36790403

[50] Choi S, Park TH, Lee HJ, et al. Subdural empyema from streptococcus suis infection, South Korea. Emerg Infect Dis. 2024;30(3):616–619. doi: 10.3201/eid3003.231018 38407167 PMC10902535

[51] Okura M, Auger JP, Shibahara T, et al. Capsular polysaccharide switching in streptococcus suis modulates host cell interactions and virulence. Sci Rep-Uk. 2021;11(1):ARTN 6513. doi: 10.1038/s41598-021-85882-3 PMC7985379 33753801

[52] Qiu X, Bai X, Lan R, et al. Novel capsular polysaccharide loci and new diagnostic tools for high-throughput capsular gene typing in streptococcus suis. Appl Environ Microbiol. 2016;82(24):7102–7112. doi: 10.1128/AEM.02102-16 27694240 PMC5118921

[53] Ye C, Zheng H, Zhang J, et al. Clinical, experimental, and genomic differences between intermediately pathogenic, highly pathogenic, and epidemic streptococcus suis. J Infect Dis. 2009;199(1):97–107. doi: 10.1086/594370 19016627

[54] Zheng H, Ye C, Segura M, et al. Mitogenic effect contributes to increased virulence of streptococcus suis sequence type 7 to cause streptococcal toxic shock-like syndrome. Clin Exp Immunol. 2008;153(3):385–391. doi: 10.1111/j.1365-2249.2008.03722.x 18803762 PMC2527368

[55] Dong XX, Chao YJ, Zhou Y, et al. The global emergence of a novel streptococcus suis clade associated with human infections. EMBO Mol Med. 2021;13(7):ARTN e13810. doi: 10.15252/emmm.202013810 PMC8261479 34137500

[56] Ngo TH, Tran TB, Tran TT, et al. Slaughterhouse pigs are a major reservoir of streptococcus suis serotype 2 capable of causing human infection in southern Vietnam. PLOS ONE. 2011;6(3):e17943. doi: 10.1371/journal.pone.0017943 21464930 PMC3065462

[57] Lavagna A, Auger JP, Giradin SE, et al. Recognition of Lipoproteins by toll-like receptor 2 and DNA by the AIM2 Inflammasome is responsible for production of interleukin-1 beta by virulent suilysin-negative streptococcus suis serotype 2. Pathogens. 2020;9(2):ARTN 147. doi: 10.3390/pathogens9020147 PMC7168628 32098284

[58] Roodsant TJ, van der Putten BCL, Tamminga SM, et al. Identification of streptococcus suis putative zoonotic virulence factors: a systematic review and genomic meta-analysis. Virulence. 2021;12(1):2787–2797. doi: 10.1080/21505594.2021.1985760 34666617 PMC8632099

[59] Xu J, Fu S, Liu M, et al. The two-component system NisK/NisR contributes to the virulence of streptococcus suis serotype 2. Microbiol Res. 2014;169(7–8):541–546. doi: 10.1016/j.micres.2013.11.002 24342108

[60] Li M, Wang C, Feng Y, et al. SalK/SalR, a two-component signal transduction system, is essential for full virulence of highly invasive streptococcus suis serotype 2. PLOS ONE. 2008;3(5):e2080. doi: 10.1371/journal.pone.0002080 18461172 PMC2358977

