# Supplementary material for: Streptococcus suis serotype 5: Emerging zoonotic threat with distinct genomic heterogeneity
Source: Virulence. 2025 Jun 26;16(1):2523882. doi: 10.1080/21505594.2025.2523882 (PMC12218517; doi:10.1080/21505594.2025.2523882)
Supplement: Supplemental Table 4.docx [file KVIR_A_2523882_SM9333.docx]

Supplemental Table 4. Pathogenic characteristics summary of *S. suis* representative strains in the study.

|  | Establishing infection | Inducing the production of pro-inflammation cytokines | Inducing organ damage | Mortality at the very early phase of infection | β-lactam resistance | Threats to public health |
| --- | --- | --- | --- | --- | --- | --- |
| 2020WUSS080 | ★★★★★ | ★★★★★ | ★★★★★ | ★★★★★ | ★★★★ | ★★★★★ |
| 2020WUSS075 | ★★★★★ | ★★★★★ | ★★★★ | ★★★★★ | ★★★★ | ★★★★★ |
| ID48908 | ★★★★★ | ★★★★★ | ★★★★ | ★★★★ | ★★★★★ | ★★★★ |
| GX169 | ★★★★ | ★★★★ | ★★★★★ | ★★★★ | ★★★★★ | ★★★★ |
| ID24665 | ★★★★ | ★★★★ | ★★★ | ★★★★ | ★★★★ | ★★★★ |
| P1/7 | ★★★★ | ★★★ | ★★★ | ★★★★ | ★★★★★ | ★★★ |
