# Supplementary material for: Streptococcus suis serotype 5: Emerging zoonotic threat with distinct genomic heterogeneity
Source: Virulence. 2025 Jun 26;16(1):2523882. doi: 10.1080/21505594.2025.2523882 (PMC12218517; doi:10.1080/21505594.2025.2523882)
Supplement: Supplemental Table 3.docx [file KVIR_A_2523882_SM9332.docx]

Supplemental Table 3. The difference in MIC value of linezolid and florfenicol between the available 42 *S. suis* serotype 5 strains from China with and without *optrA* gene.

| **Antibiotics** | **MICs (μg/mL)** | | | | | | | | **MIC_50_ (μg/mL)** | **MIC_90_ (μg/mL)** |
| --- | --- | --- | --- | --- | --- | --- | --- | --- | --- | --- |
|  | ≤0.5 | **1** | **2** | **4** | **8** | **16** | **32** | **64** |  |  |
| LZD | 23 | 5 | 10 | 4 | 0 | 0 | 0 | 0 | ≤0.5 | 2 |
| LZD of optrA positive strains | 0 | 4 | 10 | 4 | 0 | 0 | 0 | 0 | 2 | 4 |
| LZD of optrA negative strains | 23 | 1 | 0 | 0 | 0 | 0 | 0 | 0 | ≤0.5 | ≤0.5 |
| FFC | 1 | 8 | 14 | 0 | 2 | 7 | 2 | 8 | 2 | 64 |
| FFC of optrA positive strains | 0 | 0 | 0 | 0 | 1 | 7 | 2 | 8 | 32 | 64 |
| FFC of optrA negative strains | 1 | 8 | 14 | 0 | 1 | 0 | 0 | 0 | 2 | 2 |

LZD: Linezolid; FFC: Florfenicol.
