# Supplementary material for: Streptococcus suis serotype 5: Emerging zoonotic threat with distinct genomic heterogeneity
Source: Virulence. 2025 Jun 26;16(1):2523882. doi: 10.1080/21505594.2025.2523882 (PMC12218517; doi:10.1080/21505594.2025.2523882)
Supplement: Supplemental Table 2.docx [file KVIR_A_2523882_SM9331.docx]

Supplemental Table 2. The MIC values of available 42 *S. suis* serotype 5 strains from China.

| **Strains** | **Tested antimicrobial agents and breakpoints for resistant (µg/mL)** | | | | |
| --- | --- | --- | --- | --- | --- |
|  | **penicillin** | **cefotaxime** | **vancomycin** | **linezolid** | **florfenicol** |
|  | **≥1** | **≥8** | **˃1** | **˃2** | **≥8** |
|  | **MIC value of *S. suis* serotype 5 strains (µg/mL)** | | | | |
| WUSS027 | ≤0.5 | ≤0.5 | ≤0.5 | ≤0.5 | 1 |
| WUSS225 | ≤0.5 | ≤0.5 | ≤0.5 | 2 | 16 |
| WUSS233 | ≤0.5 | ≤0.5 | ≤0.5 | 1 | 16 |
| WUSS266 | ≤0.5 | ≤0.5 | ≤0.5 | 1 | 8 |
| WUSS276 | ≤0.5 | ≤0.5 | ≤0.5 | ≤0.5 | 1 |
| WUSS281 | 4 | ≤0.5 | ≤0.5 | 4 | 64 |
| WUSS289 | ≤0.5 | ≤0.5 | ≤0.5 | 1 | 32 |
| WUSS336 | ≤0.5 | ≤0.5 | ≤0.5 | ≤0.5 | 1 |
| WUSS354 | ≤0.5 | ≤0.5 | ≤0.5 | ≤0.5 | 1 |
| WUSS358 | ≤0.5 | ≤0.5 | ≤0.5 | 2 | 16 |
| WUSS363 | ≤0.5 | ≤0.5 | ≤0.5 | 2 | 64 |
| 2018WUSS006 | ≤0.5 | ≤0.5 | ≤0.5 | ≤0.5 | 1 |
| 2018WUSS036 | ≤0.5 | ≤0.5 | ≤0.5 | ≤0.5 | 2 |
| 2020WUSS051 | ≤0.5 | ≤0.5 | ≤0.5 | 1 | 8 |
| 2020WUSS075 | 1 | ≤0.5 | ≤0.5 | ≤0.5 | 1 |
| 2020WUSS080 | 1 | ≤0.5 | ≤0.5 | ≤0.5 | 2 |
| 2020WUSS085 | ≤0.5 | ≤0.5 | ≤0.5 | ≤0.5 | 2 |
| 2020WUSS088 | 1 | ≤0.5 | ≤0.5 | ≤0.5 | 2 |
| 2021WUSS081 | 4 | 4 | ≤0.5 | 4 | 32 |
| 2021WUSS082 | ≤0.5 | ≤0.5 | ≤0.5 | 2 | 64 |
| GX169 | ≤0.5 | ≤0.5 | ≤0.5 | ≤0.5 | 1 |
| YS88 | ≤0.5 | ≤0.5 | ≤0.5 | ≤0.5 | 2 |
| YS89 | ≤0.5 | ≤0.5 | ≤0.5 | ≤0.5 | 2 |
| YS106 | 2 | ≤0.5 | ≤0.5 | ≤0.5 | 2 |
| YS119 | 4 | ≤0.5 | ≤0.5 | ≤0.5 | 2 |
| YS157 | 2 | 2 | ≤0.5 | 2 | 64 |
| YS177 | ≤0.5 | ≤0.5 | ≤0.5 | 2 | 16 |
| YS188 | ≤0.5 | ≤0.5 | ≤0.5 | ≤0.5 | 2 |
| YS226 | ≤0.5 | ≤0.5 | ≤0.5 | ≤0.5 | 2 |
| YS242 | ≤0.5 | ≤0.5 | ≤0.5 | ≤0.5 | 2 |
| YS174 | ≤0.5 | ≤0.5 | ≤0.5 | ≤0.5 | 1 |
| YS259 | ≤0.5 | ≤0.5 | ≤0.5 | ≤0.5 | 2 |
| YS294 | 8 | ≤0.5 | ≤0.5 | 4 | 64 |
| YS307 | ≤0.5 | ≤0.5 | ≤0.5 | ≤0.5 | ≤0.5 |
| YS395 | 2 | ≤0.5 | ≤0.5 | 2 | 16 |
| YS468 | ≤0.5 | ≤0.5 | ≤0.5 | ≤0.5 | 2 |
| YS539 | ≤0.5 | ≤0.5 | ≤0.5 | ≤0.5 | 2 |
| YS561 | ≤0.5 | ≤0.5 | ≤0.5 | 2 | 64 |
| YS572 | ≤0.5 | ≤0.5 | ≤0.5 | 2 | 16 |
| YS580 | ≤0.5 | ≤0.5 | ≤0.5 | 2 | 16 |
| YS599 | 1 | ≤0.5 | ≤0.5 | 1 | 64 |
| YS608 | 4 | 1 | ≤0.5 | 4 | 64 |
