# Supplementary material for: Streptococcus suis serotype 5: Emerging zoonotic threat with distinct genomic heterogeneity
Source: Virulence. 2025 Jun 26;16(1):2523882. doi: 10.1080/21505594.2025.2523882 (PMC12218517; doi:10.1080/21505594.2025.2523882)
Supplement: Supplemental Table 1.docx [file KVIR_A_2523882_SM9330.docx]

Supplemental Table 1. The AR genes and AR genes associated MGEs present in the *S. suis* serotype 5 genomes.

| Lineage | | Strains | MGE site | MGE type | AR genes present in MGEs | AR genes present in out of MGE |
| --- | --- | --- | --- | --- | --- | --- |
| Lineage 1 | Lineage 1-1 | EJ2T3-2B | *rpIL* | ICE | tet(O) | erm(B) |
|  |  | EJ2T3-1B | *rpIL* | ICE | tet(O) | erm(B) |
|  |  | EJ2T3-1A | *rpIL* | ICE | tet(O) | erm(B) |
|  |  | 1607744 | *rpIL* | ICE | tet(O) | erm(B) |
|  |  | TMW_SS074 | *rpIL* | ICE | tet(O) | / |
|  |  | TMW_SS065 | *rpIL* | ICE | tet(O) | / |
|  | Lineage 1-2 | 40440 |  |  |  | erm(B), tet(M) |
|  |  | 11538 |  |  |  | None |
|  |  | GX169 | *rumA* | ICE | erm(B), ant(6)-Ia | tet(O) |
|  |  | 1547095 | *rpIL* | ICE | erm(B), tet(O) | / |
|  |  | 1652329 | *rpIL* | ICE | erm(B), tet(O) | / |
|  |  | TANI1 | *ppi* | IME | tet(O) | erm(B) |
|  |  | 93-1320 | *SSU1262* | ICE | erm(B), tet(O) | / |
|  |  | 93-2042-4514 | *SSU1262* | ICE | erm(B), tet(O) | / |
|  |  | 00-3638-4B | *SSU1262* | ICE | erm(B), tet(O) | / |
|  |  | 30212 | *SSU1262* | ICE | erm(B), tet(O) | / |
|  |  | CF2D3-4A | *ppi* | IME | tet(O) | erm(B) |
|  |  | 1148794 | *ppi* | IME | tet(O) | erm(B) |
|  |  | CF2D3-2E | *ppi* | IME | tet(O) | erm(B) |
|  |  | 92-2402-1119 |  |  |  | erm(B) |
|  |  | 1637946 |  |  |  | None |
|  |  | 90-546 | *ppi* | IME | erm(B), tet(O) | lnu(C) |
|  |  | 38728 |  |  |  | erm(B), tet(O) |
|  |  | 29898 | *ppi* | IME | erm(B), tet(O) |  |
|  | Lineage 1-3 | 2020WUSS075 | *rumA* | Prophage | erm(B), aac(6')-aph(2''), tet(40), tet(O) | lsa(E), lnu(B) |
|  |  | 2020WUSS080 | *rumA* | Prophage | erm(B), aac(6')-aph(2''), tet(40) | lsa(E) |
|  |  | ID26102 | *rpIL* | ICE | erm(B), tet(O) | ant(6)-Ib(2), tet(44), tet(W) |
|  |  | ID48908 | *rpIL* | ICE | erm(B), tet(O) | ant(6)-Ia, lsaE, lnu(B) |
|  |  | WUSS266 |  |  |  | erm(B), aph(3')-III, lsa(E), optrA*, tet(L), tet(O), mefA, lnu(B) |
|  |  | 684_10A | SSU0877 | ICE | tet(O) | erm(B) |
| Lineage 2 | Lineage 2-1 | 874 |  |  |  | erm(B), tet(O), ant(4)-Ia , ant(6)-Ia |
|  |  | 805 |  |  |  | erm(B), ant(4)-Ia , ant(6)-Ia, tet(O) |
|  |  | 803 |  |  |  | erm(B), tet(O), ant(4)-Ia , ant(6)-Ia |
|  |  | YS157 |  |  |  | erm(B), ant(6)-Ia, ant(4)-Ia , tet(O), optrA |
|  |  | YS106 | *snf2* | IME | tet(O) | erm(B), ant(6)-Ia |
|  |  | YS119 | *snf2* | IME | tet(O) | erm(B), ant(6)-Ia |
|  |  | YS395 |  |  |  | erm(B), erm(A), ant(6)-Ia, aph(3')-III, tet(O), optrA* |
|  |  | 2021WUSS081 |  |  |  | ant(6)-Ia, aac(6')-aph(2''), aph(3')-III, lsa(E), tet(O), optrA*, lnu(B) |
|  |  | YS294 |  |  |  | erm(B), aph(3')-III, ant(6)-Ia, aac(6')-aph(2''), lsa(E), tet(40), optrA, lnu(B) |
|  |  | WUSS363 |  |  |  | erm(B), ant(6)-Ia, lsa(E), tet(O/W/32/O), lnu(B) |
|  |  | YS307 |  |  |  | erm(B), ant(6)-Ia, tet(O/W/32/O) |
|  |  | YS599 | *snf2* | IME | tet(O/W/32/O) | erm(B), erm(A), optrA* |
|  |  | YS608 |  |  |  | tet(40), tet(O/W/32/O), tet(O), optrA*, cat, aac(6')-aph(2'') |
|  |  | YS88 |  |  |  | erm(B), ant(6)-Ia, tet(O) |
|  |  | YS89 |  |  |  | erm(B), ant(6)-Ia, tet(O) |
|  |  | 2021WUSS082 | *rumA* | Prophage | ant(6)-Ia, aac(6')-aph(2'') | tet(O), optrA |
|  |  | JHSJ1 |  |  |  | erm(B), ant(6)-Ia, aph(3')-III(2), lsa(E), tet(O/W/32/O), optrA*, lnu(B) |
|  |  | ZKSJ4 |  |  |  | erm(B), ant(6)-Ia, aph(3')-III(2), lsa(E), tet(O/W/32/O), optrA*, lnu(B) |
|  |  | WUSS336 |  |  |  | erm(B), ant(6)-Ia, lsa(E), tet(O/W/32/O), lnu(B) |
|  |  | YS177 |  |  |  | optrA*, tet(O), aph(3')-III, ant(6)-Ia |
|  |  | YS572 |  |  |  | ant(6)-Ia, aac(6')-aph(2''), dfrG, cat, optrA**, tet(40), tet(O) |
|  |  | YS580 |  |  |  | ant(6)-Ia, aac(6')-aph(2''), dfrG, optrA**, cat, tet(40), tet(O) |
|  |  | YS174 |  |  |  | erm(B), ant(6)-Ia, tet(40), tet(O), tet(O/W/32/O) |
|  |  | YS242 |  |  |  | erm(B), tet(O) |
|  |  | YS561 |  |  |  | erm(B), ant(6)-Ia, aph(3')-III, tet(O), optrA*, lnu(E)(2) |
|  |  | YS188 |  |  |  | tet(O) |
|  |  | YS226 |  |  |  | erm(B), tet(O), ant(6)-Ia, aac(6')-aph(2'') |
|  |  | YS259 |  |  |  | erm(B), tet(W) |
|  |  | YS468 |  |  |  | erm(B), ant(6)-Ia, aph(3')-III, tet(O), cat, lnu(B), lsa(E) |
|  | Lineage 2-2 | 2464 |  |  |  | erm(B), ant(4)-Ia, ant(6)-Ia, cfr(E), tet(O), optrA**, cat |
|  |  | 1371 |  |  |  | erm(B), ant(4)-Ia, ant(6)-Ia(2), cfr(E), tet(O), optrA**, cat |
|  |  | 180 |  |  |  | erm(B), ant(4)-Ia , ant(6)-Ia, aac(6')-aph(2''), tet(O), cat, lnu(C) |
|  |  | SH0918 | *rpIL* | ICE | erm(B)(2), cat, ant(4)-Ia , tet(O) | ant(6)-Ia(2), dfrG, optrA(2)**, cat, aac(6')-aph(2'') |
|  |  | CPD5 |  |  |  | erm(B)(2), ant(4)-Ia, ant(6)-Ia, tet(O), cat |
|  |  | CPD34 |  |  |  | erm(B), ant(4)-Ia, ant(6)-Ia, tet(O), cat |
|  |  | CPD32 |  |  |  | erm(B)(2), aph(2'')-If, ant(6)-Ia, ant(4)-Ia, tet(O), cat |
|  |  | WUSS276 |  |  |  | tet(M), ant(6)-Ia |
|  |  | 2018WUSS006 |  |  |  | erm(B), tet(M) |
|  |  | 2018WUSS036 | *rumA* | Prophage | ant(6)-Ia, aac(6')-aph(2''), tet(40), tet(O), erm(B) | tet(M) |
|  |  | WUSS354 | *snf2* | IME | erm(B), tet(O) | tet(M) |
|  |  | 2020WUSS051 |  |  |  | tet(M) |
|  |  | WUSS289 | *rumA* | Prophage | erm(B), ant(6)-Ia, aac(6')-aph(2''), cat, tet(O/W/32/O), tet(40), optrA* | / |
|  |  | 2020WUSS088 |  |  |  | erm(B), tet(M), tet(L), ant(6)-Ia |
|  |  | WUSS358 |  |  |  | ant(6)-Ia, aac(6')-aph(2''), optrA, cat, tet(L), tet(M) |
|  |  | WUSS281 |  |  |  | erm(B), aac(6')-aph(2''), ant(6)-Ia, aph(3')-III, tet(O), optrA |
|  |  | ID24665 |  |  |  | ant(6)-Ia, erm(B), tet(O), tet(O/W/32/O) |
|  |  | ID32563 | *rumA* | Prophage | tet(O/W/32/O) | erm(B), tet(O) |
|  |  | ID34567 | *rumA* | Prophage | tet(O/W/32/O) | ant(6)-Ia, tet(O), lsa(E), lnu(B) |
|  |  | WUSS225 |  |  |  | erm(B), aac(6')-aph(2''), ant(6)-Ia, tet(40), optrA**, lnu(C) |
|  |  | WUSS233 |  |  |  | erm(B), aac(6')-aph(2''), ant(6)-Ia, tet(40), optrA**, lnu(C) |
|  |  | YS539 |  |  |  | erm(B), aac(6')-aph(2''), ant(6)-Ia, dfrG, tet(M) |
|  |  | 2017UMN1435.22 | *snf2* | IME | erm(B), tet(O) | / |
|  |  | MA4T3-4A | *snf2* | IME | erm(B), tet(O) | / |
|  |  | MA4T3-4B | *snf2* | IME | erm(B), tet(O) | / |
|  |  | MA4T3-4D | *snf2* | IME | erm(B), tet(O) | / |
|  |  | 2020WUSS085 | *rumA* | ICE | erm(B), cat, tet(O), tet(L) | ant(6)-Ia(2), aac(6')-aph(2''), lsa(E), lnu(B) |
|  |  | ID41570 | *rumA* | ICE | erm(B), tet(O) | / |
|  |  | HN105 | *rpIL* | ICE | erm(B)(2), aph(3')-III, ant(6)-Ia, tet(O)(2), ant(9)-Ia | / |
|  |  | WUSS027 |  |  |  | erm(B), ant(6)-Ia, aph(3')-III |
